# Hemimetabolous genomes reveal molecular basis of termite eusociality

**DOI:** 10.1038/s41559-017-0459-1

**Published:** 2018-02-05

**Authors:** Mark C. Harrison, Evelien Jongepier, Hugh M. Robertson, Nicolas Arning, Tristan Bitard-Feildel, Hsu Chao, Christopher P. Childers, Huyen Dinh, Harshavardhan Doddapaneni, Shannon Dugan, Johannes Gowin, Carolin Greiner, Yi Han, Haofu Hu, Daniel S. T. Hughes, Ann-Kathrin Huylmans, Carsten Kemena, Lukas P. M. Kremer, Sandra L. Lee, Alberto Lopez-Ezquerra, Ludovic Mallet, Jose M. Monroy-Kuhn, Annabell Moser, Shwetha C. Murali, Donna M. Muzny, Saria Otani, Maria-Dolors Piulachs, Monica Poelchau, Jiaxin Qu, Florentine Schaub, Ayako Wada-Katsumata, Kim C. Worley, Qiaolin Xie, Guillem Ylla, Michael Poulsen, Richard A. Gibbs, Coby Schal, Stephen Richards, Xavier Belles, Judith Korb, Erich Bornberg-Bauer

**Affiliations:** 1 Institute for Evolution and Biodiversity, University of Münster, Münster, Germany.; 2 Department of Entomology, University of Illinois at Urbana-Champaign, Urbana, IL, USA.; 3 Human Genome Sequencing Center, Department of Human and Molecular Genetics, Baylor College of Medicine, Houston, TX, USA.; 4 USDA-ARS, National Agricultural Library, Beltsville, MD, USA.; 5 Evolutionary Biology & Ecology, University of Freiburg, Freiburg, Germany.; 6 Behavioral Biology, University of Osnabrück, Osnabrück, Germany.; 7 Ecology and Evolution, University of Copenhagen, Copenhagen, Denmark.; 8 Institute of Science and Technology Austria, Klosterneuburg, Austria.; 9 Institut de Biologia Evolutiva, CSIC-University Pompeu Fabra, Barcelona, Spain.; 10 Department of Entomology and Plant Pathology, North Carolina State University, Raleigh, NC, USA.; 11 China National GeneBank, Beijing Genomics Institute (BGI)-Shenzhen, Shenzhen, China.; 12 These authors contributed equally: Mark C. Harrison, Evelien Jongepier and Hugh M. Robertson.

## Abstract

Around 150 million years ago, eusocial termites evolved from within the *cockroaches*, 50 million years before eusocial Hymenoptera, such as bees and ants, appeared. Here, we report the 2-Gb genome of the German cockroach, *Blattella germanica*, and the 1.3-Gb genome of the drywood termite *Cryptotermes secundus*. We show evolutionary signatures of termite eusociality by comparing the genomes and transcriptomes of three termites and the cockroach against the background of 16 other eusocial and non-eusocial insects. Dramatic adaptive changes in genes underlying the production and perception of pheromones confirm the importance of chemical communication in the termites. These are accompanied by major changes in gene regulation and the molecular evolution of caste determination. Many of these results parallel molecular mechanisms of eusocial evolution in Hymenoptera. However, the specific solutions are remarkably different, thus revealing a striking case of convergence in one of the major evolutionary transitions in biological complexity.

Eusociality, the reproductive division of labour with overlapping generations and cooperative brood care, is one of the major evolutionary transitions in biology^1^. Although rare, eusociality has been observed in a diverse range of organisms, including shrimps, mole rats and several insect lineages^2–4^. A particularly striking case of convergent evolution occurred within the holometabolous Hymenoptera and in the hemimetabolous termites (Isoptera), which are separated by over 350 Myr of evolution^5^. Termites evolved within the cockroaches around 150 Myr ago, towards the end of the Jurassic period^6,7^, about 50 Myr before the first bees and ants appeared5. Therefore, identifying the molecular mechanisms common to both origins of eusociality is crucial to understanding the fundamental signatures of these rare evolutionary transitions. While the availability of genomes from many eusocial and non-eusocial hymenopteran species^8^ has allowed extensive research into the origins of eusociality within ants and bees^9–11^, a paucity of genomic data from cockroaches and termites has precluded large-scale investigations into the evolution of eusociality in this hemimetabolous clade.

The conditions under which eusociality arose differ greatly between the two groups. Termites and cockroaches are hemimetabolous and so show a direct development, while holometabolous hymenopterans complete the adult body plan during metamorphosis. In termites, workers are immatures and only reproductive castes are adults^12^, while in Hymenoptera, adult workers and queens represent the primary division of labour. Moreover, termites are diploid and their colonies consist of both male and female workers, and usually a queen and king dominate reproduction. This is in contrast to the haplodiploid system found in Hymenoptera, in which all workers and dominant reproductives are female. It is therefore intriguing that strong similarities have evolved convergently within the termites and the hymenopterans, such as differentiated castes and a nest life with reproductive division of labour. The termites can be subdivided into wood-dwelling and foraging termites. The former belong to the lower termites and produce simple, small colonies with totipotent workers that can become reproductives. Foraging termites (some lower and all higher termites) form large, complex societies, in which worker castes can be irreversible^12^. For this reason, higher, but not lower, termites can be classed as superorganismal^13^. Similarly, within Hymenoptera, varying levels of eusociality exist.

Here we provide insights into the molecular signatures of eusociality within the termites. We analysed the genomes of two lower and one higher termite species and compared them to the genome of the German cockroach, *Blattella germanica*, as a closely related non-eusocial outgroup. Furthermore, differences in expression between nymphs and adults of the cockroach were compared to differences in expression between workers and reproductives of the three termites, to gain insights into how expression patterns changed along with the evolution of castes. Using 15 additional insect genomes to infer background gene family turnover rates, we analysed the evolution of gene families along the transition from non-social cockroaches to eusociality in the termites. In this study, we concentrated particularly on two hallmarks of insect eusociality, as previously described for Hymenoptera, with the expectation that similar patterns occurred along with the emergence of termites. These are the evolution of a sophisticated chemical communication, which is essential for the functioning of a eusocial insect colony^3,14,15^, and major changes in gene regulation along with the evolution of castes^9,10^. We also tested whether transposable elements spurred the evolution of gene families that were essential for the transition to eusociality.

## Evolution of genomes, proteomes and transcriptomes

**Fig. 1:**
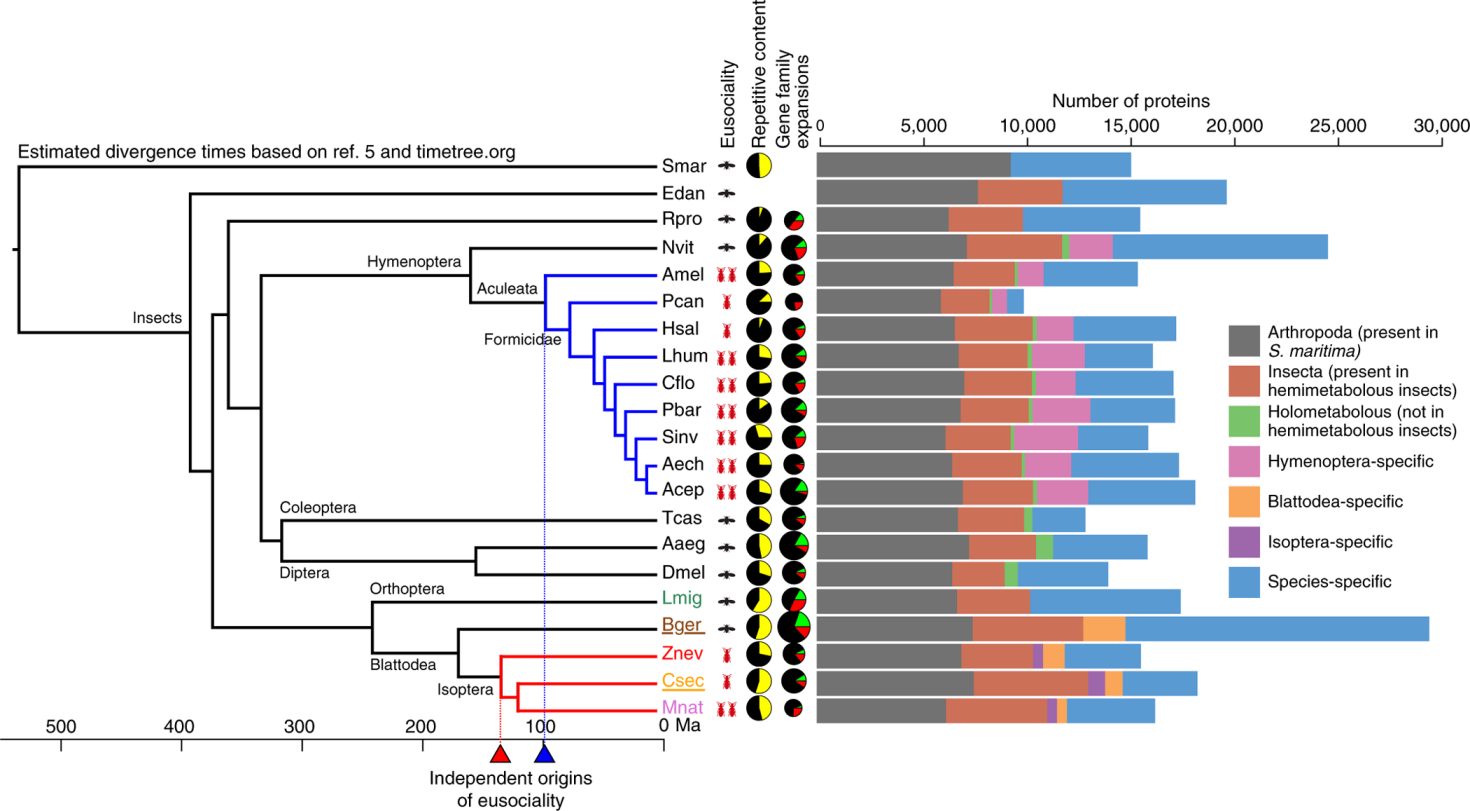
Phylogenetic, genomic and proteomic comparisons of 20 insect species. From left to right: a phylogenetic tree of 20 insect species with *Strigamia maritima* (centipede) as the outgroup (species of newly sequenced genomes presented in this study are underlined); level of eusociality (one red insect: simple eusociality; two red insects: advanced eusociality; black fly: non-eusocial); fractions of repetitive content (yellow) within genomes of selected species (for sources, see Supplementary Information); proportions of species-specific gene family expansions (green), contractions (red) and stable gene families (black), the size of the pies represents the relative size of the gene family change (based on total numbers); a bar chart showing protein orthology across taxonomic groups within each genome. Ma, million years ago. Smar, *Strigamia maritima*; Edan, *Ephemera danica*; Rpro, *Rhodnius prolixus*; Nvit, *Nasonia vitripennis*; Amel, *Apis mellifera*; Pcan, *Polistes canadensis*; Hsal, *Harpegnathos saltator;* Lhum, *Linepithema humile*; Cflo, *Camponotus floridanus*; Pbar, *Pogonomyrmex barbatus;* Sinv, *Solenopsis invicta*; Aech, *Acromyrmex echinatior*; Acep, *Atta cephalotes*; Tcas, *Tribolium castaneum*; Aaeg, *Aedes aegypti*; Dmel, *Drosophila melanogaster*; Lmig, *Locusta migratoria*; Bger, *Blattella germanica*; Znev, *Zootermopsis nevadensis*; Csec, *Cryptotermes secundus*; Mnat, *Macrotermes natalensis*.

**Fig. 2:**
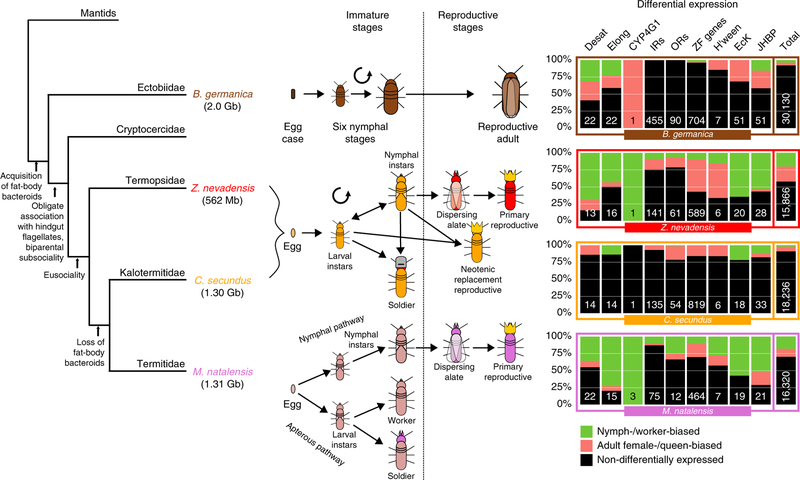
Comparison of developmental pathways between *B*. *germanica*, the lower termites *Z*. *nevadensis* and *C*. *secundus*, and the higher termite *M*. *natalensis*. Shown from left to right are: a simple phylogeny^97^ describing important novelties along the evolutionary trajectory to termites (numbers in brackets are genome sizes); life cycles; differential expression (log_2_(fold change) > 1 and *P* < 0.05; DESeq2^79^; sample sizes are shown in the last column) between workers and queens (between nymphs and adult females in *B*. *germanica*) of the selected gene families (Desat, desaturases; Elong, elongases; H’ween, Halloween genes) and total numbers within all genes; the numbers denote total numbers of genes in each gene family.

We sequenced and assembled the genome of the German cockroach, *B*. *germanica* (Ectobiidae), and of the lower, drywood termite *Cryptotermes secundus* (Kalotermitidae; for assembly statistics, see Supplementary Table 1). The cockroach genome (2.0 Gb) is considerably larger than all three termite genomes. The genome size of *C*. *secundus* (1.30 Gb) is comparable to the higher, fungus-growing termite *Macrotermes natalensis* (1.31 Gb, Termitidae)^16^, but more than twice as large as the lower, dampwood termite *Zootermopsis nevadensis* (562 Mb, Termopsidae)^17^. The smaller genomes of termites compared to the cockroach are in line with previous size estimations based on C-values^18^. The proteome of *B*. *germanica* (29,216 proteins) is also much larger than in the termites, where we find the proteome size in *C*. *secundus* (18,162) to be similar to those of the other two termites (*M*. *natalensis*: 16,140; *Z*. *nevadensis*: 15,459; Fig. 1). In fact, the *B*. *germanica* proteome was the largest among all 21 arthropod species analysed here (Fig. 1). Strong evi-dential support for over 80% of these proteins in *B*. *germanica* (see Supplementary Information) and large expansions in many manually annotated gene families offer high confidence in the accuracy of this proteome size. We also compared gene expression between the four species. When comparing worker expression with queen expression in the termites and nymph expression (fifth and sixth instars) with adult female expression in *B*. *germanica*, we found shifts in specificity of expression for termites compared to the cockroach in several gene families (Fig. 2). It has previously been reported for the primitively eusocial paper wasp *Polistes canadensis* that the majority of caste-biased genes, especially those upregulated in workers, are novel genes^19^. The authors suggested that this may be a feature of early eusociality. We did not find the same pattern for the termites. Species-specific genes (those without an orthologue) were not enriched for differentially expressed genes in any of the termites, with slight peaks among Blattodea- and Isoptera-specific genes (Supplementary Fig. 1).

## Gene family expansions assisted by TEs in termites

The transitions to eusociality in ants^10^ and bees^9^ have been linked to major changes in gene family sizes. Similarly, we detected significant gene family changes on the branch leading to the termites (seven expansions and ten contractions; Supplementary Fig. 2 and Supplementary Table 2). The numbers of species-specific, significant expansions and contractions of gene families varied within termites (*Z*. *nevadensis*: 15/5; *C*. *secundus*: 27/3; *M*. *natalensis*: 24/20; Supplementary Fig. 2 and Supplementary Tables 3–5). Interestingly, in *B*. *germanica* we measured 93 significant gene family expansions but no contractions (Supplementary Table 6), which contributed to the large proteome.

The termite and cockroach genomes contain a higher level of repetitive DNA compared to the hymenopterans we analysed (Fig. 1). *C*. *secundus* and *B*. *germanica* genomes both contain 55% repetitive content (Supplementary Table 7), which is higher than in both *Z*.*nevadensis*(28%)andthehighertermite*M*.*natalensis*(46%;Fig. 1)^20^. As also found in *Z*. *nevadensis* and *M*. *natalensis*^20^, LINEs and especially the subfamily BovB were the most abundant transposable elements (TEs) in the *B*. *germanica* and *C*. *secundus* genomes, indicating that a proliferation of LINEs may have occurred in the ancestors of Blattodea (cockroaches and termites).

We hypothesized that these high levels of TEs may be driving the high turnover in gene family sizes within the termites and *B*. *germanica*^21^. Expanded gene families indeed had more repetitive content within 10-kb flanking regions in all three termites (*P* < 1.3 × 10^−8^; Wald *t*-test; Supplementary Tables 8 and 9), in particular in the higher termite *M*. *natalensis*. In contrast, gene family expansions were not correlated with TE content in flanking regions for *B*. *germanica*. These results suggest that a major expansion of LINEs at the root of the Blattodea clade contributed to the evolution of gene families within termites, probably via unequal crossing-over^21^; however, the expansions in *B*. *germanica* were not facilitated by TEs. It can therefore be speculated that the large expansion of LINEs within Blattodea allowed the evolution of gene families that ultimately facilitated the transition to eusociality.

## Expansion and positive selection of ionotropic receptors

**Fig. 3:**
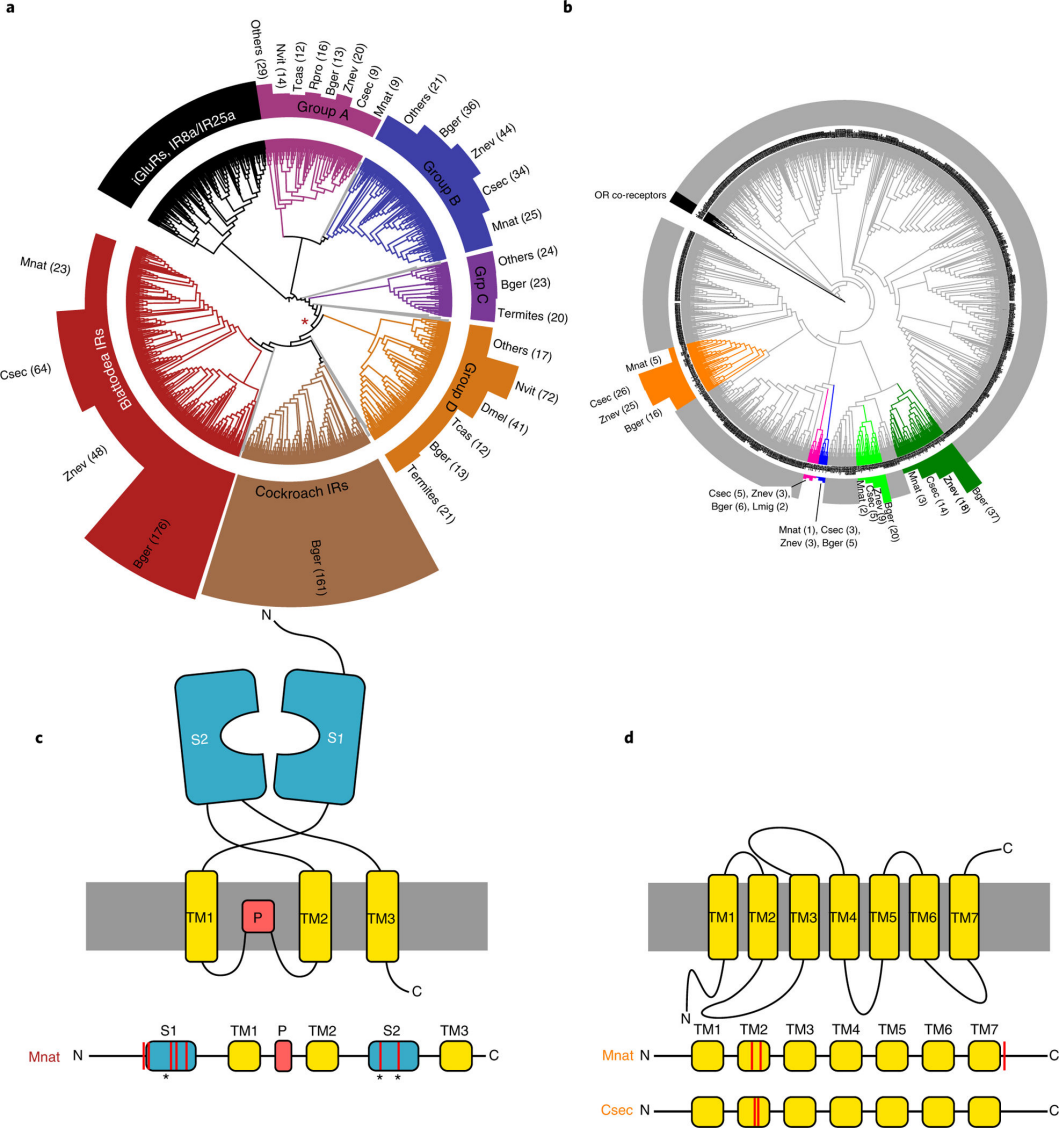
Expansions, contractions and positive selection within iRs and ORs in termites. **a**,**b**, IR (**a**) and OR (**b**) gene trees of 13 insect species. In each tree, only well-supported clades (support values > 85) that include *B*. *germanica* or termite genes are highlighted within the gene trees. The lengths of the coloured bars represent the number of genes per species within each of these clades. The red asterisk in **a** denotes the putative root of intronless IRs. **c**, The upper schematic diagram depicts the 2D structure of an IR, containing ligand-binding lobes (S1 and S2), transmembrane regions (TM1–3) and the pore domain (P). Below, the sequence of the domains along the peptide is represented, showing that the sites, which are under significant positive selection (red bars; codeml site models 7 and 8) within Blattodea IRs for *M*. *natalensis* (*P* < 1.7 × 10^−10^; likelihood-ratio test, 5 sequences, 413 aligned codons), are all situated within the ligand-binding lobes and on or around the putative ligand-binding sites (asterisks)^86^. **d**, The same representation for ORs, which include eight transmembrane regions. Positive selection was found for *M*. *natalensis* (*P* = 1.1 × 10^−10^; 5 sequences, 1,001 aligned codons) and *C*. *secundus* (*P* = 5.6 × 10^−16^; likelihood ratio test, 26 sequences, 1,913 aligned codons) of the orange clade, each at two codon positions within the second transmembrane region and at a third position within the carboxy-terminal extracellular region for *M*. *natalensis.*

Insects perceive chemical cues from toxins, pathogens, food and pheromones with three major families of chemoreceptors, the odorant (ORs), gustatory and ionotropic (IRs) receptors^22^. ORs, in particular, have been linked to colony communication in eusocial Hymenoptera, where they abound^14,15,23^. Interestingly, as previously detected for *Z*. *nevadensis*^17^, the OR repertoire is sub-stantially smaller in *B*. *germanica* and all three termites compared to hymenopterans. IRs, on the other hand, which are less frequent in hymenopterans, are strongly expanded in the cockroach and termite genomes (Fig. 3 and Supplementary Fig. 3). Intronless IRs, which are known to be particularly divergent^24^, show the greatest cockroach- and Blattodea-specific expansions (Fig. 3a, Blattodea-, Cockroach- and Group D-IRs). By far the most IRs among all investigated species were found in *B*. *germanica* (455 complete gene models), underlining that the capacity for detecting many differ- ent kinds of chemosensory cues is crucial for this generalist that thrives in challenging, human environments. In line with a special- ization in diet and habitat, the total number of IRs is lower within the termites (*Z*. *nevadensis*: 141; *C*. *secundus*: 135; *M*. *natalensis*: 75). Nevertheless, IRs are more numerous in termites than in all other analysed species (except *Nasonia vitripennis*: 111). This is strik- ingly similar to the pattern for ORs in Hymenoptera, which are also highly numerous in non-eusocial outgroups as well as in eusocial sister species^14,23,25^.

We scanned each IR group for signs of species-specific positive selection. Within the Blattodea-specific intronless IRs, we found two codon positions under significant selection for the higher termite *M*. *natalensis* (codeml site models 7 and 8; *P* = 5.4 × 10^−5^). Within a subgroup of five sequences, this was more strongly pronounced with seven codon positions under significant positive selection for *M*. *natalensis* (*P* < 1.7 × 10^−10^). The positively evolving codons are situated within the two ligand-binding lobes of the receptors (Fig. 3c), showing that a diversification of ligand specificity has occurred along with the transition to higher eusociality and a change from wood-feeding to fungus-farming in *M*. *natalensis*. Only two IRs were differentially expressed between nymphs and adult females in *B*. *germanica*. Underlining a change in expression along with the evolution of castes, we found 35 IRs to be differentially expressed between workers and queens in *Z*. *nevadensis*, 11 in *C*. *secundus* and 10 in *M*. *natalensis* (Fig. 2 and Supplementary Table 10). The possible role of IRs in pheromonal communication has been highlighted both in the cockroach *Periplaneta americana*^26^ and in *Drosophila melanogaster*^27^, where several IRs show sex-biased expression.

One group of ORs (orange clade in Fig. 3b) is evolving under significant positive selection at codon positions within the second transmembrane domain in *M*. *natalensis* (codeml site model; *P* = 1.1 × 10^−11^) and *C*. *secundus* (*P* = 5.6 × 10^−16^; Fig. 3d). Such a variation in the transmembrane domain can be related to ligand-binding specificity, as has been shown for a polymorphism in the third transmembrane domain for an OR in *D*. *melanogaster*^28,29^, adding further support for an adaptive evolution of chemoreceptors, in line with the greater need for a sophisticated colony communication in the termites. Similar to IRs, a higher proportion of ORs were differentially expressed between workers and queens in the three termites than between nymphs and adults in the cockroach (Fig. 2 and Supplementary Table 11), highlighting a change in expression and function along with the transition to eusociality. The evolution of chemoreceptors along with the emergence of the termites can also be related to adaptation processes and changes in diet compared to the cockroach. Experimental verification will help pinpoint which receptors are particularly important for communication.

## CHC-producing enzymes have evolved caste-specificity

Despite their different ancestry, both termites and eusocial hymenopterans are characterized by the production of caste- specific cuticular hydrocarbons (CHCs)^30–32^, which are often crucial for regulating reproductive division of labour and chemical communication. Accordingly, we find changes in the termites in three groups of proteins involved in the synthesis of CHCs: desaturases (introduction of double bonds^33^), elongases (extension of C-chain length^34^) and CYP4G1 (last step of CHC biosynthesis^35^).

Desaturases are thought to be important for division of labour and social communication in ants^36^. As previously described for ants^36^, Desat B genes are the most abundant desaturase family in the termites and the cockroach (Supplementary Table 12), especially in *M*. *natalensis* where we found ten gene copies (significant expansion; *P* = 0.0003; Supplementary Table 5 and Supplementary Figure 4). As in ants, especially the first desaturases (Desat A–Desat E) vary greatly in their expression between castes and species in the three termites (Fig. 2 and Supplementary Table 13)^36^. In contrast to ants, where these genes are under strong purifying selection^36^, for the highly eusocial termite *M*. *natalensis*, we found significant positive selection within the Desat B genes (codeml site models 7 and 8; *P* = 1.1 × 10^−16^), indicating a diversification in function, possibly related to their greater diversification of worker castes (major and minor workers, major and minor soldiers). Although desaturases are often discussed in the context of CHC production and chemical communication, their biochemical roles are quite diverse^36^, and the positive selection we observe for *M*. *natalensis* may, at least in part, be related to their rather different ecology of foraging and fungus-farming rather than nest-mate recognition. Future experimental verification of the function of these genes will help better understand these observed genomic and transcriptomic patterns.

Underlining an increased importance of CHC communication in termites, the expression patterns of elongases (extension of C-chain length) differ considerably in the termites compared to the cockroach (Fig. 2 and Supplementary Table 14). In contrast to *B*. *germanica*, in which elongases are both nymph- (five genes) and adult-biased (four genes), only one or two elongase genes in each termite are queen-biased in their expression, while many are worker-biased. As with the desaturases, a group of *M*. *natalensis* elongases also reveal significant signals of positive selection (codeml branch- site test; *P* = 4 × 10^−4^), further indicating a greater diversification of CHC production in this higher termite.

The last step of CHC biosynthesis, the production of hydrocarbons from long-chain fatty aldehydes, is catalysed by a P450 gene, *CYP4G1*, in *D*. *melanogaster*^35^. We found one copy of *CYP4G1* in *B*. *germanica*, *Z*. *nevadensis* and *C*. *secundus*, but three copies in *M*. *natalensis*, reinforcing the greater importance of CHC synthesis in this higher termite. Corroborating the known importance of maternal CHCs in *B*. *germanica*^37^, CYP4G1 is overexpressed in female adults compared to nymphs (Fig. 2 and Supplementary Table 15). In each of the termites, however, CYP4G1 is more highly expressed in workers (or kings in *C*. *secundus*) compared to queens (Fig. 2 and Supplementary Table 15), adding support that, com- pared to cockroach nymphs, a change in the dynamics and turnover of CHCs in termite workers has taken place.

## Changes in gene regulation in termites

**Fig. 4:**
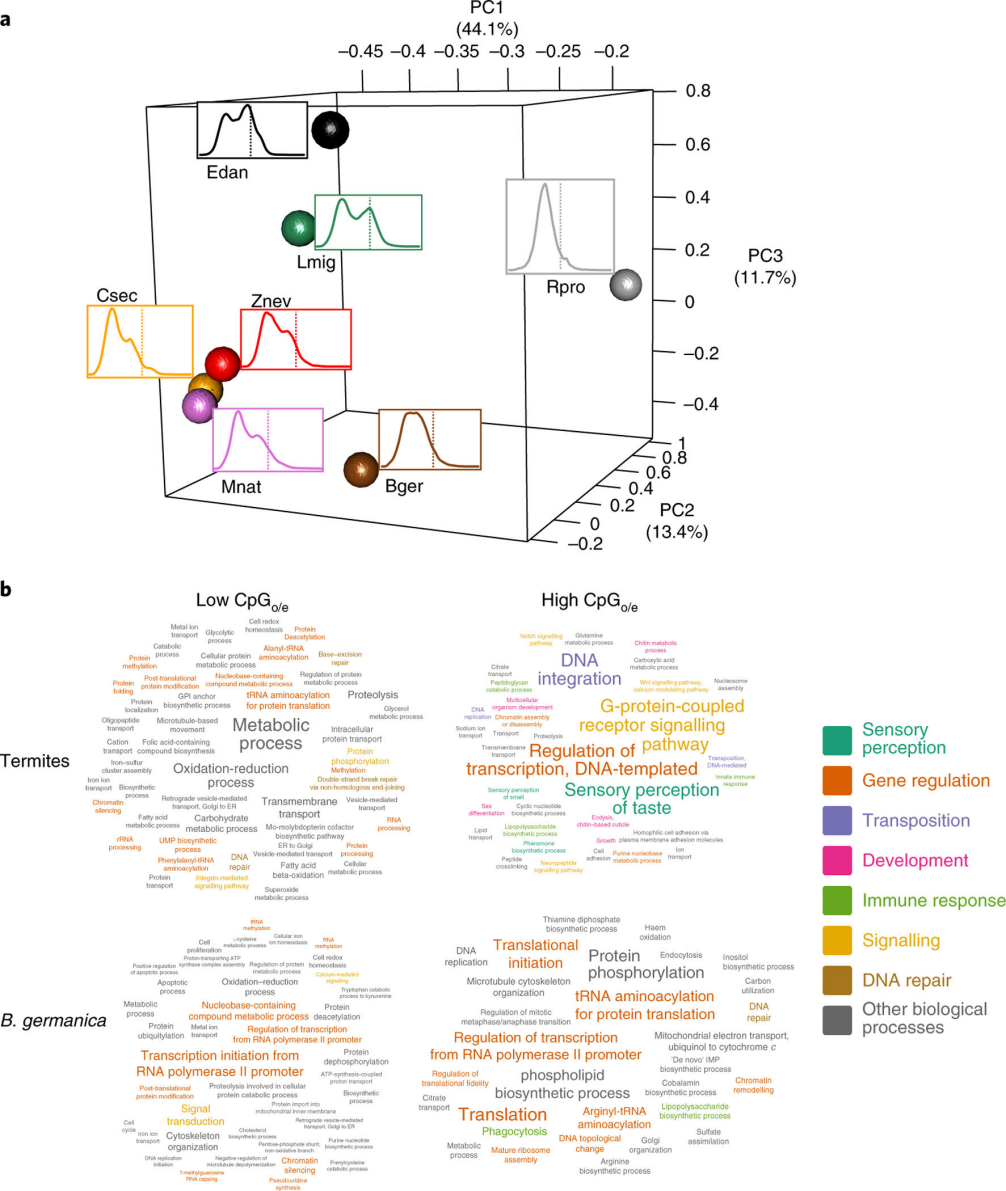
CpG_o/e_ of seven hemimetabolous insects. **a**, Principal component analysis (PCA) of predicted DNA methylation patterns among 2,664 1-to-1 orthologues, estimated via CpG_o/e_. The spheres represent the positions of the species within the 3D PCA, with the distance between the spheres representing the similarity of CpG_o/e_ between species at each orthologue; the curves are the distribution of CpG_o/e_, with the dotted line showing CpG_o/e_ = 1. **b**, Tag clouds of enriched (*P* < 0.05; Fisher test, weight algorithm, topGO^96^) GO terms (biological processes) among the lower (left) and the higher quartile (right) of CpG_o/e_ within termites (top) and *B*. *germanica* (bottom). For termites, genes were merged from all three species for analysing GO term enrichment. Number of enriched genes and total number of genes in topGO enrichment tests (low CpG*o/e*/high CpG_o/e_/gene universe): *B*. *germanica* (3,291/1,842/11,409); termites (6,754/4,600/25,910). High CpG_o/e_ indicates a low level of DNA methylation and vice versa.

The development of distinct castes underlying division of labour is achieved via differential gene expression. Major changes in gene regulation have been reported as being central to the transition to eusociality in bees^9^ and ants^10^. Accordingly, we found major changes in putative DNA methylation patterns (levels per 1-to-1 orthologue) among the termites compared to four other hemimetabolous insect species (Fig. 4a). This is revealed by CpG depletion patterns (CpG_o/e_, observed versus expected number of CpGs), a reliable predictor of DNA methylation^38,39^, correlating more strongly between the termites than among any of the other analysed hemimetabolous insects (Fig. 4). In other words, within orthologous genes, predicted DNA methylation levels differ greatly between termites and other hemimetabolous species but remain conserved among termite species.

The predicted levels of DNA methylation correlated negatively with the caste-specificity of expression for each of the termites. This is confirmed by a positive correlation between CpG_o/e_ (negative association with level of DNA methylation) and absolute log_2_(fold change) of expression between queens and workers (Pearson’s *r* = 0.32 to 0.36; *P* < 2.2 × 10^−16^). The caste-specific expression of putatively unmethylated genes in termites is reflected in the enrichment of GO terms related to sensory perception, regulation of transcription, signalling and development, whereas methylated genes are mainly related to general metabolic processes (Fig. 4b and Supplementary Table 16). These results show strong parallels to findings for eusocial Hymenoptera^40–43^. This is in stark contrast to the non-eusocial cockroach, *B*. *germanica*, where there was only a very weak relationship between CpG_o/e_ and differential expression between nymphs and adult females (*r* = 0.14), nor were any large differences apparent in enriched GO terms between putatively methylated and non-methylated genes (Fig. 4b).

Our results argue in favour of a diminished role of DNA methylation in caste-specific expression within eusocial insects, as recently shown^38,44^. In fact, DNA methylation appears to be important for the regulation of housekeeping genes because predicted methylated genes are related to general biological processes (further supported by lower CpG_o/e_ within 1-to-1 orthologues than in non-conserved genes)^45^, while caste-specific genes are ‘released’ from this type of gene regulation. However, a recent study linked caste-specific DNA methylation to alternative splicing in *Z*. *nevadensis*^46^.

Major biological transitions are often accompanied by expansions of transcription factor (TF) families, such as genes containing zinc-finger (ZF) domains^47^. We also observed large differences in ZF families within the termites compared to *B*. *germanica*. Many ZF families were reduced or absent in termites, while different, unrelated ZF gene families were significantly expanded (Supplementary Tables 2–6). Queen-biased genes were significantly over-represented among ZF genes for each of the termites (*P* < 2 × 10^−10^; *χ*^2^ test; Supplementary Table 17), indicating an important role of ZF genes in the regulation of genes related to caste-specific tasks and colony organization in the termites. This is in contrast to the significant under-representation of differentially expressed ZF genes within *germanica* (*P* = 4.8 × 10^−5^; *χ*_2_-test). Interestingly, two other important TF families (bHLH and bZIP)^47^, which were not expanded in the termites, showed no caste-specific expression pattern (*P* > 0.05), except bZIP genes, in which queen-biased genes were marginally over-represented for *M*. *natalensis* (*P* = 0.049). These major upheavals in ZF gene families and their caste-specific expression show that major changes in TFs accompanied the evolution of termites, strikingly similar to the evolution of ants^10^.

## Evolution of genes related to moulting and metamorphosis

Hemimetabolous eusociality is characterized by differentiated castes, which represent different developmental stages. This is in contrast to eusocial Hymenoptera, in which workers and reproductives are adults. While cockroaches develop directly through several nymphal stages before becoming reproductive adults, termite development is more phenotypically plastic, and workers are essentially immatures (Fig. 2). In wood-dwelling termites, such as *C*. *secundus* and *Z*. *nevadensis*, worker castes are non-reproductive immatures that are totipotent to develop into other castes, while in the higher termite *M*. *natalensis*, workers can be irreversibly defined instars. It is therefore clear that a major change during the evolution of termites occurred within developmental pathways. Accordingly, we found changes in expression and gene family size of several genes related both to moulting and metamorphosis.

In the synthesis of the moulting hormone, 20-hydroxyecdysone, the six Halloween genes (five cytochrome P450s and a Rieske-domain oxygenase) play a key role^48,49^. Only one Halloween gene, Shade (Shd; CYP314A1), which mediates the final step of 20-hydroxyecdysone synthesis, is differentially expressed between the final nymphal stages and adult females in *B*. *germanica* (Fig. 2 and Supplementary Table 18), consistent with its role in the nymphal or imaginal moult. In the three termites, the Halloween genes show varying caste-specific expression (Fig. 2 and Supplementary Table 18), showing that ecdysone plays a significant role in the regulation of caste differences. Ecdysteroid kinase genes (EcK), which con- vert the insect moulting hormone into its inactive state, ecdysone 22-phosphate, for storage^50^, are only overexpressed in female adults compared to nymphs in *B*. *germanica* (16/51 genes, Fig. 2 and Supplementary Table 19). In termites, however, where the gene copy number is reduced (18 to 20 per species), these important moulting genes appear to have evolved worker-specific functions (Fig. 2 and Supplementary Table 19).

Whereas 20-hydroxyecdysone promotes moulting, juvenile hormone (JH) represses imaginal development in pre-adult instars^51^. JH is important in caste differentiation in eusocial insects, including termites^12,52^. Haemolymph JH-binding proteins (JHBPs), which transport JH to its target tissues^53^, are reduced within the termites (21 to 33 genes) but significantly expanded in *B*. *germanica* (51 copies; *P* = 0018; Supplementary Table 6). Thirteen of the JHBP genes are overexpressed in adult females and only 8 in nymphs in *B*. *germanica* (Fig. 2 and Supplementary Table 20). In both *Z*. *nevadensis* and *M*. *natalensis*, on the other hand, JHBPs are significantly more worker-biased (*P* < 0.01, *χ*^2^ test; Supplementary Table 20 and Fig. 2). In *C*. *secundus*, expression is more varied, with four worker-biased, seven king-biased and two queen-biased genes (Fig. 2 and Supplementary Table 20).

These changes in copy number and caste-specific expression of genes involved in moulting and metamorphosis within termites compared to the German cockroach demonstrate that changes occurred in the control of the developmental pathway along with the evolution of castes. However, this interpretation needs to be experimentally verified.

## Conclusions

These results, considered alongside many studies on eusociality in Hymenoptera^9, 10, 14,36^, provide evidence that major changes in gene regulation and the evolution of sophisticated chemical communication are fundamental to the transition to eusociality in insects. Strong changes in DNA methylation patterns correlated with broad-scale modifications of expression patterns. Many of these modified expression patterns remained consistent among the three studied termite species and occurred within protein pathways essential for eusocial life, such as CHC production, chemoperception, ecdysteroid synthesis and JH transport. The stronger patterns we observe for *M*. *natalensis*, especially within genes linked to chemical communication, such as the expansion of Desat B and CYP4G1 genes and significant positive selection in desaturases, elongases and IRs, may be associated with this termite’s higher level of eusociality and its status as a superoganism^13^. The analysis of further higher and lower termites would shed light on the generality of these patterns and possibly assist in the distinction between the influences of eco- logical and eusocial traits.

Many of the mechanisms implicated in the evolution of eusociality in the termites occurred convergently around 50 Myr later in the phylogenetically distant Hymenoptera. However, several details are unique due to the distinct conditions within which eusociality arose. One important difference is the higher TE content within cockroaches and termites, which probably facilitated changes in gene family sizes, supporting the transition to eusociality. However, the most striking difference is the apparent importance of IRs for chemical communication in the termites, compared to ORs in Hymenoptera. According to our results, the non-eusocial ancestors of termites possessed a broad repertoire of IRs, which favoured the evolution of important functions for colony communication in these chemoreceptors within the termites, whereas in the solitary ancestors of eusocial hymenopterans ORs were most abundant^14, 25^. The parallel expansions of different chemoreceptor families in these two independent origins of eusociality indicate that convergent selection pressures existed during the evolution of colony communication in both lineages.

## Methods

### Genome sequencing and assembly.

Genomic DNA from a single *Blattella germanica* male from an inbred line (strain: American Cyanamid = Orlando Normal) was used to construct two paired-end (180-bp and 500-bp inserts) and one of the two mate-pair libraries (2-kb inserts). An 8-kb mate-pair library was constructed from a single female. The libraries were sequenced on an Illumina HiSeq2000 sequencing platform. The 413 Gb of raw sequence data were assembled with Allpaths LG^54^, and then scaffolded and gap-filled using the in-house tools Atlas-Link v.1.0 (https://www.hgsc.bcm.edu/software/atlas-link) and Atlas gap-fill v.2.2. For *Cryptotermes secundus*, three paired-end libraries (250-bp, 500-bp and 800-bp inserts) and three mate-pair libraries (2-kb, 5-kb and 10-kb inserts) were constructed from genomic DNA that was extracted from the head and thorax of 1,000 individuals, originating from a single, inbred field colony. The libraries were sequenced on an Illumina HiSeq2000 sequencing platform. The *C*. *secundus* genome was assembled using SOAPdenovo (v.2.04)^55^ with optimized parameters, followed by gapcloser (v1.10, released with SOAPdenovo) and kgf (v1.18, released with SOAPdenovo).

### Transcriptome sequencing and assembly.

For annotation purposes, 22 whole- body RNA-sequencing (RNA-Seq) samples from various developmental stages were obtained for *B*. *germanica*. For *C*. *secundus*, RNA-Seq libraries were obtained for three workers, four queens and four kings, based on degutted, whole-body extracts. In addition, we sequenced ten *Macrotermes natalensis* RNA-Seq libraries from three queens, one king and six pools of workers. All libraries were constructed using the Illumina (TruSeq) RNA-Seq kit.

For protein-coding gene annotation, *B*. *germanica* reads were assembled with de novo Trinity (version r2014–04-13)^56^. The *C*. *secundus* reads were assembled using Cufflinks on reads mapped with TopHat (version2.2.1)^57,58^, de novo Trinity^56^ and genome-guided Trinity on reads mapped with TopHat.

### Repeat annotation.

A custom *C*. *secundus* and *B*. *germanica* repeat library was constructed using a combination of homology-based and de novo approaches, including RepeatModeler/RepeatClassifier (http://www.repeatmasker.org/RepeatModeler/), LTRharvest/LTRdigest^59^ and TransposonPSI (http://transposonpsi.sourceforge.net/). The ab initio repeat library was complemented with the RepBase (update 29 August 2016)^60^ and SINE repeat databases, filtered for redundancy with CD-hit and classified with RepeatClassifier. RepeatMasker (version open-4.0.6, http://www.repeatmasker.org) was used to mask the *C secundus* and *B*. *germanica* genome. Repeat content for the other studied species (Fig. 1) was obtained from the literature^61–67^.

### Protein-coding gene annotation.

The *B*. *germanica* genome was annotated with Maker (version 2.31.8)^68^, using the species-specific repeat library, *B*. *germanica* transcriptome data (22 whole-body RNA-Seq samples) and the Swiss-Prot/ UniProt database (last accessed: 21 January 2016) plus the *C*. *secundus* and *Zootermopsis nevadensis* protein sequences for evidence-based gene model predictions. AUGUSTUS (version 3.2)^69^, GeneMark-ES Suite (version 4.21)^70^ and SNAP^71^ were used for ab initio predictions. *C. secundus* protein-coding genes were predicted using homology-based, *ab initio* and expression-based methods, and integrated into a final gene set (see Supplementary Information). Gene structures were predicted by GeneWise^72^. The ab initio annotations were predicted with AUGUSTUS^73^ and SNAP^71^, retained if supported by both methods and integrated with the homology-based predictions using GLEAN^74^. Transcriptome-based gene models were merged with PASA^75^ and tested for coding potential with CPC^76^ and OrfPredictor^77^. PASA gene models were merged with the homology-based and *ab initio* gene set, retaining the PASA models in case of overlap. Desaturases, elongases, chemosensory receptors, cytochrome P450s and genes involved in the juvenile hormone pathway were manually curated in Blattodea.

### Differential gene expression.

The *C*. *secundus* and *M*. *natalensis* RNA-Seq libraries were complemented with nine published *Z*. *nevadensis* libraries, yielding two to six libraries from workers, queens and kings for each termite. These were compared to six of the *B*. *germanica* libraries: two from fifth instar nymphs, two from sixth instar nymphs and two from adult females. Reads were mapped to the genome using HiSat2^78^. Read counts per gene were obtained using htseq-count and DESeq2^79^ was used for differential expression analysis. Differential expression analysis between kings (males), queens (females) and workers (majors and minors combined for *M*. *natalensis*) was assessed for the termites. For *B*. *germanica* we evaluated the differential expression between adults and the two last nymphal stages combined, with the assumption that the final nymphal stages are homologous to termite workers and the adult females are homologous to termite queens. Genes were considered significantly differentially expressed if *P* < 0.05 and log_2_(fold change) >|1| in order to account for allometric differences as recommended in a previous study^80^.

### Protein orthology.

In addition to *B. germanica*, *C. secundus*, *Z. nevadensis* and *M. natalensis*, 16 other insect proteomes were included in our analyses: *Locusta migratoria*, *Rhodnius prolixus*, *Ephemera danica*, *Drosophila melanogaster*, *Aedes aegypti*, *Tribolium castaneum*, *Nasonia vitripennis*, *Polistes canadensis*, *Apis mellifera*, *Harpegnathos saltator*, *Linepithema humile*, *Camponotus floridanus*, *Pogonomyrmex barbatus*, *Solenopsis invicta*, *Acromyrmex echinatior* and *Atta cephalotes*; as well as for the centipede *Strigamia maritima* as an outgroup (for sources, see Supplementary Table 22). These proteomes were grouped into orthologous clusters with OrthoMCL^81^, with a granularity of 1.5.

### IR and OR identification, phylogeny and structure.

Ionotropic receptors (IRs) were identified using two custom hidden Markov models (HMMs) obtained with hmmbuild and hmmpress of the HMMER suite^82^. The first HMM comprises the IR’s ion channel and ligand-binding domain based on a MAFFT^83^ protein alignment of 76 IRs from 15 species (Supplementary Table 23). The second HMM was built to distinguish IRs from iGluRs, IR8a and IR25a, which have an additional amino-terminal domain^24^. For this we built an HMM from 48 protein sequences (Supplementary Table 23). The proteomes were scanned with pfam_scan and the two custom HMMs, where proteins that matched the IR HMM, but not the amino- terminal domain HMM were annotated as IRs. Odorant receptors (ORs) were identified on the basis of the Pfam domain PF02949 (7tm OR).

Multiple sequence alignments of IRs and ORs were obtained with hmmalign^82^, using the Pfam OR HMM PF02949 and custom IR HMM to guide the alignment. Gene trees were computed with FastTree^84^ (options: -pseudo -spr 4 –mlacc 2 -slownni) and visualized with iTOL v3^85^. Putative IR ligand-binding residues and structural regions were identified on the basis of the alignments with *melanogaster* IRs and iGluRs of known structure^86^.

### Gene family expansions and contractions.

For the analyses of gene family expansions and contractions, the hierarchical clustering algorithm MC-UPGMA^87^ was used, with a ProtoLevel cutoff of 80 (ref. ^88^). Protein families were further divided into sub-families if they contained more than 100 proteins in a single species, or more than an average of 35 proteins per species. Proteins were blasted against the RepeatMasker TE database (E-value < 10^−5^) and clusters where > 50% of the proteins were identified as transposable elements were discarded. Clade- and species-specific protein family expansions and contractions, were identified with CAFE v3.0^89^ using the same protocol as in previous studies^9,10^ (see also Supplementary Information).

### TE-facilitated expansions.

The repeat content in the 10-kb flanking regions of *B*. *germanica*, *C*. *secundus*, *Z*. *nevadensis* and *M*. *natalensis* genes was calculated using bedtools^90^. Coding DNA sequences (CDSs) from neighbouring genes were removed and the repeat content was analysed using generalized linear mixed models (glmmPQL implemented in the R^91^ package MASS^92^) with binomial error distribution. Fixed predictors included gene family expansion, species ID and their interaction. Cluster ID was fitted as a random factor to avoid pseudo-replication. Significance was assessed on the basis of the Wald *t-*test (R package aod^93^) at *α* < 0.05. Main and interaction effects for each of the genomic regions are listed in Supplementary Table 8. Model parameters are listed in Supplementary Table 8.

### Tests for positive selection.

To test for positive selection within gene families of interest, site model tests 7 and 8 were performed (model = 0; NSsites = 7 8) on species-specific CDS alignments, or branch-site test (model = 2; NSsites = 2; fix_ omega = 1 for null model and 0 for alternative model) on multi-species alignments. Protein sequences were aligned using MAFFT^83^ with the E-INS-i strategy, and CDS alignments were created using pal2nal.pl^94^. Phylogenetic trees were created with FastTree^84^. Alignments were trimmed using Gblocks (settings: -b2 = 21; -b3 = 20; -b4 = 5; -b5 = a). Models were compared using likelihood-ratio test and where *P* < 0.05, Bayes empirical Bayes results were consulted for codon positions under positive selection (*P* < 0.05).

### CpG depletion patterns and GO enrichment.

To estimate DNA methylation, we compared observed to expected CpG counts within CDS sequences^38,39^. A low CpG_o/e_ indicates a high level of DNA methylation, as the cytosines of methylated CpGs often mutate to thymines. Expected CpG counts were calculated by dividing the product of cytosine and guanine counts by the sequence length. The principal component analysis in Fig. 4 was created using the R function prcomp on log- transformed CpG_o/e_ values for all 1-to-1 orthologues for the seven hemimetabolous species. These orthologues were extracted from the OrthoMCL results. The three-dimensional (3D) plot was created with the plot3d command from the R package rgl.

CpG-depleted (first quartile) and -enriched (fourth quartile) genes were tested for enrichment of Gene Ontology terms. Pfam protein domains were obtained for *germanica*, *Z*. *nevadensis*, *C*. *secundus* and *M*. *natalensis* protein sequences using PfamScan^95^. Corresponding GO terms were obtained with Pfam2GO. GO-term over-representation was assessed using the TopGO^96^ package in R. Enrichment analysis was performed using the weight algorithm selecting nodesize = 10 to remove terms with fewer than ten annotated GO terms. After that, GO terms classified as significant (topGOFisher < 0.05) were visualized using the R package tagcloud (https://cran.r-project.org/web/packages/tagcloud/).

## Supplementary Material

supplemental
