# Supplementary material for: Hemimetabolous genomes reveal molecular basis of termite eusociality
Source: Nat Ecol Evol. Author manuscript; Available in PMC 2019 Apr 25. (PMC6482461; doi:10.1038/s41559-017-0459-1)
Supplement: supplemental [file NIHMS1022840-supplement-supplemental.pdf]

nature ecology & evolution

SUPPLEMENTARY INFORMATION
https://doi.org/10.1038/s41559-017-0459-1

In the format provided by the authors and unedited.

OPEN

Hemimetabolous genomes reveal molecular basis of termite eusociality

Mark C. Harrison[1,12], Evelien Jongepier[1,12], Hugh M. Robertson[2,12], Nicolas Arning[1], Tristan Bitard-Feildel[1], Hsu Chao[3], Christopher P. Childers[4], Huyen Dinh[3], Harshavardhan Doddapaneni[3], Shannon Dugan[3], Johannes Gowin[5,6], Carolin Greiner[5,6], Yi Han[3], Haofu Hu[7], Daniel S. T. Hughes[3], Ann-Kathrin Huylmans[8], Carsten Kemena[1], Lukas P. M. Kremer[1], Sandra L. Lee[3], Alberto Lopez-Ezquerra[1], Ludovic Mallet[1], Jose M. Monroy-Kuhn[5], Annabell Moser[5], Shwetha C. Murali[3], Donna M. Muzny[3], Saria Otani[7], Maria-Dolors Piulachs[9], Monica Poelchau[4], Jiaxin Qu[3], Florentine Schaub[5], Ayako Wada-Katsumata[10], Kim C. Worley[3], Qiaolin Xie[11], Guillem Ylla[9], Michael Poulsen[7], Richard A. Gibbs[3], Coby Schal[10], Stephen Richards[3], Xavier Belles[9*], Judith Korb[5,6*] and Erich Bornberg-Bauer[1*]

[1]Institute for Evolution and Biodiversity, University of Münster, Münster, Germany. [2]Department of Entomology, University of Illinois at Urbana-Champaign, Urbana, IL, USA. [3]Human Genome Sequencing Center, Department of Human and Molecular Genetics, Baylor College of Medicine, Houston, TX, USA. [4]USDA-ARS, National Agricultural Library, Beltsville, MD, USA. [5]Evolutionary Biology & Ecology, University of Freiburg, Freiburg, Germany. [6]Behavioral Biology, University of Osnabrück, Osnabrück, Germany. [7]Ecology and Evolution, University of Copenhagen, Copenhagen, Denmark. [8]Institute of Science and Technology Austria, Klosterneuburg, Austria. [9]Institut de Biologia Evolutiva, CSIC-University Pompeu Fabra, Barcelona, Spain. [10]Department of Entomology and Plant Pathology, North Carolina State University, Raleigh, NC, USA. [11]China National GeneBank, Beijing Genomics Institute (BGI)-Shenzhen, Shenzhen, China. [12]These authors contributed equally: Mark C. Harrison, Evelien Jongepier and Hugh M. Robertson.
*e-mail: xavier.belles@ibe.upf-csic.es; judith.korb@biologie.uni-freiburg.de; ebb@uni-muenster.de

© 2018 Macmillan Publishers Limited, part of Springer Nature. All rights reserved.

Contents

MATERIALS & METHODS 3

1 Genome sequencing and assembly 3

1.1 *Blattella germanica* genome . . . 3

1.2 *Cryptotermes secundus* genome . . . 3

2 Protein-coding gene annotation 4

2.1 *Blattella germanica* . . . 4

2.2 *Cryptotermes secundus* . . . 4

2.3 Additional manual annotations . . . 5

3 Genome quality assessment 5

4 Transcriptomes 6

4.1 Transcriptome assembly . . . 6

4.2 Phylostratigraphy . . . 6

5 Species datasets and protein orthology 6

5.1 Elongases . . . 7

6 Chemosensory receptors 7

6.1 Identification of IRs and ORs . . . 7

6.2 Gene tree and structural analysis of IRs and ORs . . . 7

7 Gene family expansions and contractions 8

7.1 Clustering proteins into families . . . 8

7.2 Finding family expansions and contractions . . . 9

SUPPLEMENTARY FIGURES 11

SUPPLEMENTARY TABLES 17

REFERENCES 56

MATERIALS & METHODS

1 Genome sequencing and assembly

1.1 *Blattella germanica* genome

Blattella germanica is one of thirty arthropod species sequenced as a part of a pilot project for the i5K arthropod genomes project at the Baylor College of Medicine Human Genome Sequencing Center. For all of these species, an enhanced Illumina-ALLPATHS-LG[1] sequencing and assembly strategy enabled multiple species to be approached in parallel at reduced costs. For most species, including *B. germanica* the pilot, we sequenced four libraries of nominal insert sizes 180bp, 500bp, 3kb and 8kb. The amount of sequence generated from each of these libraries is noted in table S21. The 180bp, 500bp and 3kb mate pair libraries were made from a single male individual, and the 8kb mate pair library from genomic DNA of a single female.

To prepare the 180bp and 500bp libraries, we used a gel-cut paired-end library protocol. Briefly, 1 μg of the DNA was sheared using a Covaris S-2 system (Covaris, Inc. Woburn, MA) using the 180-bp or 500-bp program. Sheared DNA fragments were purified with Agencourt AMPure XP beads, end-repaired, dA-tailed, and ligated to Illumina universal adapters. After adapter ligation, DNA fragments were further size selected by agarose gel and PCR amplified for 6 to 8 cycles using Illumina P1 and Index primer pair and Phusion® High-Fidelity PCR Master Mix (New England Biolabs). The final library was purified using Agencourt AMPure XP beads and quality assessed by Agilent Bioanalyzer 2100 (DNA 7500 kit) determining library quantity and fragment size distribution before sequencing.

The long mate pair libraries with 3kb or 8kb insert sizes were constructed according to the manufacturer's protocol (Mate Pair Library v2 Sample Preparation Guide art # 15001464 Rev. A PILOT RELEASE). Briefly, 5 μg (for 2 and 3-kb gap size library) or 10 μg (8-10 kb gap size library) of genomic DNA was sheared to desired size fragments by Hydroshear (Digilab, Marlborough, MA), then end repaired and biotinylated. Fragment sizes between 3-3.7 kb (3 kb) or 8-10 kb (8 kb) were purified from 1% low melting agarose gel and then circularized by blunt-end ligation. These size selected circular DNA fragments were then sheared to 400 bp (Covaris S-2), purified using Dynabeads M-280 Streptavidin Magnetic Beads, end-repaired, dA-tailed, and ligated to Illumina PE sequencing adapters. DNA fragments with adapter molecules on both ends were amplified for 12 to 15 cycles with Illumina P1 and Index primers. Amplified DNA fragments were purified with Agencourt AMPure XP beads. Quantification and size distribution of the final library was determined before sequencing as described above. Sequencing was performed on Illumina HiSeq2000s generating 100 bp paired-end reads. Illumina reads were pre-processed with SeqPrep (`https://github.com/jstjohn/SeqPrep`). Reads from mate-pair libraries were separated into forward and reverse using Cutadapt.[2]

For *B. germanica* the 2.0 Gb of raw sequence data were assembled into 24 820 scaffolds with a scaffold N50 of 1 056 Kb, 317 391 contigs with a contig N50 of 12 126 bp, and a sequence coverage of 158x (Table S1). The *B. germanica* genome size was estimated at 2.0 Gb, based on the 17-mer depth distribution.

1.2 *Cryptotermes secundus* genome

Genomic DNA was extracted from the head and thorax from 1 000 individuals, originating from a single, inbred field colony. To obtain DNA of sufficient quality, the extractions were done in aliquots, comprising 5-6 individuals each, following a modified CTAB protocol as described in.[3] Thus, we obtained about 200

extractions which were quality checked and then pooled to obtain 7 samples. Six of the 7 samples were of high enough quality to be used for library construction. These included three short insert, paired-end libraries (250 bp, 500 bp and 800 bp inserts) and three mate pair libraries (2 kb, 5 kb and 10 kb inserts). Libraries were sequenced on the Illumina HiSeq2000 sequencing platform. Prior to assembly, several filtering steps were applied to remove:

- reads with more than 5% 'N'-bases or polyA,
- reads with more than 50 low-quality bases (Phred score $\leq$7),
- reads with adapter sequences,
- paired reads with more than 10bp overlap (allowing 10% mismatches) and
- PCR duplicates with identical paired-end reads.

The *C. secundus* genome was assembled using SOAPdenovo (v.2.04)[4] with optimised parameters. gap-closer (v1.10, released with SOAPdenovo) and kgf (v1.18, released with SOAPdenovo) were used to fill gaps in the assembly. We also checked if there were any contaminated sequences in the assembly. The sequences of the assembly were searched against the NCBI nt database of bacteria and fungi by BLASTN, but no long and high-score blast hits were found. Thus, the assembly is unlikely to have contaminated sequences.

For *C. secundus* we had 1.0 Gb of raw sequence data, which were assembled into 55 493 scaffolds with a scaffold N50 of 1 184 Kb, 89 222 contigs with a contig N50 of 63 937 bp, and a sequence coverage of 45x (Table S1). The *C. secundus* genome size was estimated at 1.3 Gb based on the 17-mer depth distribution.

2 Protein-coding gene annotation

2.1 *Blattella germanica*

The *B. germanica* genome was annotated using a 2-pass, iterative Maker (version 2.31.8)[5] workflow, which masks repeat elements, aligns transcriptome data and protein predictions to the genome, produces *ab initio* gene predictions and synthesises evidence into final annotations. Three *ab initio* gene modellers were applied: AUGUSTUS (version 3.2),[6] pre-trained on 2 675 highly conserved, single-copy Arthropod orthologs (i.e. Busco Arthropod data base);[7] GeneMark-ES Suite (version 4.21),[8] run in self-training mode; and Snap.[9]

For evidence-based gene model predictions, we included (i) the species-specific repeat library (see Materials and Methods of main text), (ii) *B. germanica* transcriptome data (see section 4) and (iii) a protein set composed of the swissprot/uniprot data base (last accessed: 21-01-2016), and the *C. secundus* and *Zootermopsis nevadensis* protein predictions.

2.2 *Cryptotermes secundus*

Protein-coding genes were predicted based on homology, *ab initio* predictions and expression data; and integrated into a final gene set. Protein sequences from 12 species were used in homology-based predictions: *Apis mellifera*, *Caenorhabditis elegans*, *Drosophila melanogaster*, *Homo sapiens Atta cephalotes*, *Acromyrmex echinatior*, *Camponotus floridanus*, *Harpegnathos saltator*, *Linepithema humile*, *Pogonomyrmex barbatus* and the two termites *Zootermopsis nevadensis* and *Macrotermes natalensis*. Protein sequences were aligned to the *C. secundus*assembly by TBLASTN. For each aligned region the most

similar homolog was selected if $>$ 50% of the query protein's length. Gene structures were predicted by GeneWise.[10] To reduce false positives, only predictions with a CDS length $>$ 150bp and a GeneWise score $>$ 50 were retained. *Ab initio* annotation was done with AUGUSTUS[11] and SNAP,[9] using 500 randomly selected genes with complete ORFs from homology-based annotations of *A. mellifera* for training. To reduce false positives, only *ab initio* predictions that were supported by both AUGUSTUS and SNAP were retained. Transcript-based annotations (see section 4 for details on transcriptome assembly) were merged with PASA[12] and tested for coding potential with CPC.[13] Candidate coding genes were investigated with OrfPredictor[14] and the resulting potential CDSs were mapped onto the transcripts to predict UTRs.

The homology-based, transcript-based and *ab initio* annotations were merged with GLEAN.[15] Where gene models overlapped, the transcriptome-based PASA gene models were kept. Further well supported annotations were added based on the following criteria: 1) homology-based predictions with complete ORFs and GeneWise scores $>$ 80; 2) transcript-based predictions with complete ORFs; and 3) *ab initio* predictions with a putative SwissProt function. Putative transposable elements according to the Interpro and SwissProt were discarded from the final annotation.

To exclude scaffolds of prokaryotic origin, the *B. germanica* and *C. secundus* preliminary annotations were blasted against the NCBI non-redundant database (last accessed: 29-07-2014). Scaffolds where $>$75% of the genes had prokaryotic best blast hits were labelled as contaminant and excluded from the final annotation.

2.3 Additional manual annotations

Some gene families implicated in the evolution of eusociality, were manually curated in the four Blattodea species (*B. germanica*, *Z. nevadensis*, *C. secundus*, *M. natalensis*). These include genes encoding desaturases (Figs. S4,S5,S6, tables S12,S13), elongases (Table S14), chemosensory receptors (Figure S3) and Cytochrome P450's (Table S15), as well as those involved in juvenile hormone synthesis. The annotation of chemosensory receptors are detailed in section 6.1.The other families were manually annotated using a pipeline developed in-house called `geneSearch` that includes information from different sources. `geneSearch` proposes candidate genes based on orthology information computed with `OrthoMCL` (v2.0.9).[16] BLAST results and domain content based on Pfam v30.0[17] annotation are included as well as additional information, such as coverage and similarity. Based on this information the genes belonging to a gene family were chosen. In case of possible erroneous gene models `Augustus` (3.1.0)[11] using hints produced by `exonerate` (2.2.0)[18] was used to improve gene models. Gene trees were then calculated using `calcTree`, a small pipeline and part of `geneSearch`. It computes an MSA using `T-COFFEE` (10.00.r1613),[19] that is trimmed in the next step using `trimAl` (1.4).[20] `RAXML` (7.2.8)[21] is then used to reconstruct the phylogeny. To determine the correct parameters for `RAXML`, `prottest`[22] (v.3.4) is used. The trees are visualised using `showTree`, a Python script that uses `ETE3`.[23]

3 Genome quality assessment

The quality and contiguity of the *C. secundus* and *B. germanica* genomes were evaluated using Busco (version 2.0),[7] Dogma (version 2.0)[24] and Quast (version 3.1).[25] Busco assesses the completeness of the assembly based on a set of 2 675 one-to-one orthologs that are widely conserved among Arthropods. Dogma evaluates the completeness of the annotation, based on common insect protein domains and domain arrangements.

Despite the high gene count in *B. germanica* (Table S1), gene annotations were generally well supported by the underlying evidence: 83.4% of the *B. germanica* genome annotations had an Annotation Edit Distance (AED) score < 0.5, indicative of a well annotated genome. Moreover, 82.01% of the annotated genes had >75% of their length covered by EST support or protein alignments. In total, 79.7 and 81.8% of the conserved Arthropod genes had complete orthologs in the *B. germanica* and *C. secundus* assemblies, respectively. Similarly, Dogma reported 78.14 and 88.38% completeness of the respective gene annotations. The completeness of the *B. germanica* and *C. secundus* genomes is thus comparable to those of the other two termite species (Table S1).

4 Transcriptomes

4.1 Transcriptome assembly

For the *B. germanica* genome annotation, 22 RNAseq paired-end libraries of different developmental stages were obtained for genome annotation, a subset of which was included in the differential gene expression analyses (section **??**). Libraries were sequenced on the Illumina MiSeq platform, reads assembled using Trinity (version r2014-04-13)[26] at default settings and transcripts filtered for redundancy using CD-HIT (version 4.6; default settings).[27] *Cryptotermes secundus* paired-end RNAseq libraries were obtained for three workers (larval instars, without wing buds), four queens (reproducing primary females) and four kings (reproducing primary males). In all cases, whole body extracts (without guts) were used. Libraries were constructed using the Illumina (TruSeq) RNA-Seq kit and sequenced on the Illumina HiSeq2000 platform. The *C. secundus* RNAseq reads were assembled using i) Cufflinks on reads mapped with TopHat (version2.2.1),[28,29] ii) *de novo* Trinity (version r2014-04-13);[26] and iii) genome-guided Trinity on reads mapped with TopHat. In addition, 11 RNAseq libraries were obtained for *Macrotermes natalensis*, for differential expression analyses (see section **??**). Samples included three queens, six pools of major and minor workers and two kings. We obtained 75bp long, paired-end reads which were sequenced on the Illumina NextSeq 500 platform.

4.2 Phylostratigraphy

A phylostratigraphy approach as described by[30] was performed to classify termite and *B. germanica* proteins in different age groups using blastp. Using this strategy the genes were classified in several ages: PS1_Mandibulata (older genes), PS2_Pterygota, PS3_Neoptera, PS4_Orthopteroidea, PS5_Blattoidea, PS6_Isoptera and PS7_Species-specific. The proportions of differentially expressed genes within each age class were then calculated and compared to previous results for *Polistes canadensis*.[31] See figure S1 and main text for further details.

5 Species datasets and protein orthology

In addition to the *C. secundus* and *B. germanica* protein predictions, protein annotations were obtained for 18 other insect species: *Macrotermes natalensis*, *Zootermopsis nevadensis*, *Locusta migratoria*, *Rhodnius prolixus*, *Ephemera danica*, *Drosophila melanogaster*, *Aedes aegypti*, *Tribolium castaneum*, *Nasonia vitripennis*, *Apis mellifera*, *Polistes canadensis*, *Harpegnathos saltator*, *Linepithema humile*, *Camponotus floridanus*, *Pogonomyrmex barbatus*, *Solenopsis invicta*, *Acromyrmex echinatior* and *Atta cephalotes*; as

well as for the centipede, *Strigamia maritima*, as an outgroup. The online sources of these files are listed in Table S22. Sequences containing a stop codon in the middle of a sequence were removed (exceptions were made for known selenoproteins). Only the longest isoforms were included in our analyses. These proteomes were grouped into homologous clusters with OrthoMCL,[32] using default settings. For the MCL clustering, a granularity of 1.5 was chosen.

5.1 Elongases

Elongases were identified by the ELO domain (PF01151). In total we found 22 elongases in *B. germanica*, 14 in *C. secundus*, 16 in *Z. nevadensis* and 15 in *M. natalensis* (Figure S14).

6 Chemosensory receptors

6.1 Identification of IRs and ORs

To assess the size variation of the olfactory receptor repertoire across the phylogeny, we identified ionotropic receptors (IRs) and odorant receptors (ORs) based on sequence motifs within 13 insect proteomes (table S22, species marked with †). ORs were identified with the Pfam domain `PF02949` (7tm Odorant receptor). Since we found no Pfam domain that matched IRs exclusively and consistently, we built two custom hidden Markov models (HMMs) using `hmmbuild` and `hmmpress` of the HMMER suite (see additional data file 10).[33] The first HMM comprises the ion channel and ligand-binding domain of IRs. This HMM is based on a MAFFT[34] protein alignment of 76 IRs from 15 species (Table S23a). The second HMM was built to distinguish IRs from iGluRs, IR8a and IR25a. iGluRs and IR8a/IR25a have an additional amino-terminal domain (ATD)[35] for which we built an HMM from 48 protein sequences (Table S23b). The proteomes were scanned with `pfam_scan` and the two custom HMMs. Proteins that matched the IR HMM, but not the ATD HMM were considered IRs. This method of IR identification was tested on the *Drosophila grimshawi* proteome,[36] a species that was not used for HMM building and thus represents a fair test dataset. Fifty-three *D. grimshawi* IRs were identified which is very close to the 52 annotated *D. grimshawi* IRs reported in.[35] Similarly, we found 141 *Z. nevadensis* IRs which is close to the number of 137 IRs that were reported in.[37] We note, however, that this domain-based IR annotation assumes a complete genome annotation and may thus underestimate the true number of IRs in some species.

Due to the extremely high number of IRs in *B. germanica* and the close synteny of many, these methods were deemed insufficient for the annotation of olfactory receptors in *B. germanica*. Therefore, ORs and IRs of *B. germanica* were manually annotated using methods similar to those for the damp-wood termite *Z. nevadensis*,[37] and using the proteins from that analysis as queries in TBLASTN searches, with E-values of 1000. Iterative searches with newly identified genes/proteins were conducted exhaustively. Gene models were built in the WebApollo browser available at the i5k WorkSpace at the National Agriculture Library (`https://i5k.nal.usda.gov/`), as best possible. Occasionally, the genome assembly was repaired using raw reads from the genome project as well as RNAseq from antennae and heads (C. Schal, unpublished). The number of identified IRs and ORs in thirteen insect species is shown in Figure S3.

6.2 Gene tree and structural analysis of IRs and ORs

The protein sequence of ligand-binding IRs and ORs is highly variable, even within the same species.[35,38] This variability and the high number of sequences makes them difficult to align. To overcome these issues,

we first restricted the analysed species to a set of thirteen representative species (table S22, species marked with †). IRs and ORs were then aligned separately using `hmmalign` of the HMMER suite.[33] `hmmalign` aligns sequences to an HMM profile and then outputs a multiple sequence alignment. This approach makes it possible to align proteins that are structurally conserved, even if they diverge strongly in their sequence. The OR alignment was guided by the Pfam OR HMM `PF02949` and the IR alignment was guided by our custom IR HMM. Gene trees were computed with FastTree[39] using pseudocounts (`-pseudo` option, recommended for gappy alignments) and parameters for an exhaustive, accurate tree reconstruction (`-spr 4 -mlacc 2 -slownni`). Gene trees were visualised with iTOL v3.[40] We used colours to highlight sufficiently supported (branch support > 0.85) IR and OR groups containing Blattodea proteins.

Sites under positive selection were identified with codeml of the PAML suite[41] as described in Materials & Methods of the main text. Putative ligand-binding residues and structural regions (S1,S2,M1,M2,M3,P) of IRs were reported in.[42] These regions and residues were identified in two *M. natalensis* IR groups (Figure 2B of the main text) by aligning them with *D. melanogaster* IRs and iGluRs of known structure.[42]

7 Gene family expansions and contractions

7.1 Clustering proteins into families

The OrthoMCL protein clustering (see section 5) produces small clusters of highly similar proteins. This is ideal for the identification of one-to-one orthologs. However, the term "protein family" is usually used to describe a larger group of proteins that often consists of multiple paralogs per species. To achieve a more coarse-grained protein clustering, the hierarchical clustering algorithm MC-UPGMA[43] was used. MC-UPGMA was developed to identify protein families in huge datasets and is used by the ProtoNet database.[44] The protein identity matrix of twenty arthropod proteomes (same as in section 5) was used as the basis for MC-UPGMA clustering. The matrix was converted to the required input format: a gzipped, tab-separated text file. This file contains three values per row: two protein IDs and their similarity score (BLAST E-value). Additionally, the following pre-processing steps were required by MC-UPGMA:

- each protein ID was replaced with a unique numerical ID
- hits with a BLAST E-value above 10 were discarded
- hits of a protein against itself ($i \leftrightarrow i$) were discarded
- redundant edges were discarded (i.e. only one of $i \leftrightarrow j$ and $j \leftrightarrow i$)
- if there were multiple BLAST hits (local alignments) between two proteins, the lowest E-value was selected

MC-UPGMA clustering was executed with the command

```
$ perl ./mcupgma_1.0.0/scripts/cluster.pl x.edges.gz -max_singleton 320670 -x 100.0 -M 800000000
 ↪  -H 70 -K 69 -j 69 -r 0 -iterations 10000 -sleep 1 -tree x.mcupgma_tree
```

where `x.edges.gz` is the gzipped input file, `320670` is the highest numerical protein ID that was used and `x.mcupgma_tree` is the hierarchical clustering output file. The remaining parameters denote the available computational resources. For convenience and compatibility, `x.mcupgma_tree` was converted to orthoMCL output format and numerical IDs were mapped back to the original protein IDs. Since MC-UPGMA is a hierarchical clustering algorithm, it does not simply group proteins into families. Instead, it determines one or more tree-like hierarchies that connect proteins based in their similarity. To retrieve protein families from

this hierarchy, ProtoNet introduced the ProtoLevel measure. The ProtoLevel can be used as a cut-off to divide the protein hierarchy into protein families. A high cut-off yields large, diverse protein families and a low cut-off yields small families of closely related proteins. A ProtoLevel cut-off of 80 was used. Additionally, protein families were further divided into sub-families if they contained more than 100 proteins in a single species, or more than an average of 35 proteins per species. This step is recommended in the CAFE v3.0 manual, since very large gene families can cause CAFE[45] to die during computation.

7.2 Finding family expansions and contractions

CAFE v3.0[45] was used to determine clade- and species-specific protein family expansions and contractions based on the MC-UPGMA gene family clusters. CAFE estimates the rate of gains and losses per gene per million years (the gene birth/death parameter λ) across the phylogeny. A p-value is then calculated for each gene family, which indicates whether the family deviates significantly from the neutral birth/death null model. Significant family p-values indicate adaptive family size evolution.[45] Such families are then subjected to a detailed analysis where the significance of node to node size transitions is determined. This detailed analysis can reveal species- or clade-specific gene family expansions and contractions.

MC-UPGMA determined 16 208 gene families in total. Transposable element families (1246 in total) were discarded since their extreme size variability had a strong influence on further analyses, as recommended in.[46] A family was considered a TE gene family when more than half of the proteins had a significant (E-value $< 10^{-5}$) BLASTp hit against the RepeatMasker TE database. A limitation of CAFE is that it cannot model family size evolution if the family was not yet present in the last common ancestor (LCA) of the species. This means that very young gene families cannot be screened for expansions or contractions. To increase the number of usable families, the two most basal arthropods *S. maritima* and *E. danica* were excluded from this analysis. Families that were not present in the LCA of the 19 remaining insect species were discarded with CAFE's `-filter` option.

```
tree ((((nvi:162,(ame:97,(pca:76,((((((aec:9,ace:9):9,sin:18):9,pba:27):10,cfl:37):9,lhu:46):9,
hsa:55):21):21):65):182,(tca:327,(aae:158,dme:158):169):17):29,rpr:373):14,((((mna:121,cse:121)
:15,zne:136):37,bge:173):75,lmi:248):139)
```

By default, CAFE models family size evolution with a single birth/death rate λ for the whole phylogeny. To obtain a more accurate null model that allows for different birth/death rates at different branches, it is possible to specify a "λ-structure" with the option `lambda -t`. This option can specify branches, or groups of branches that should receive a separate λ estimate. Since the fully parametrised model (an independent λ for each branch, 36 λ in total) did not converge, we used the same approximation as[46] and:[47] The 36 branches were clustered into groups that evolve at a similar birth/death rate. Branches were clustered based on a two-column matrix: The first column is the birth/death rate of the foreground branch and the second column is the shared birth/death rate of all background branches. These values were estimated with a two-parameter (foreground λ and background λ) run for each branch.

Branches were clustered using k-means clustering with k values ranging from 3 to 20. This resulted in 18 different branch models, in addition to four models that we defined manually:

- model 1: one global λ rate for all branches of the phylogeny
- model 2: a global gene gain rate and a global gene loss rate (λ/μ-model)
- model 3: two λ rates, one for eusocial branches and one for non-eusocial branches
- model 4: three λ rates, one each for non-eusocial, primitive eusocial and advanced eusocial branches

- models k_3 to k_{20}: 3 – 20 λ rates, based on k-means clustering as described above

To ensure that each model converged to the global maximum, we executed every CAFE script five times. Model k_{16} (see Figure S2) best fit the data (likelihood ratio test). Expansions or contractions were considered significant when CAFE's Viterbi branch p-value was below 0.01 and the family p-value was below the default threshold of 0.05. Expanded and contracted families are listed for the branch leading up to Isoptera (Table S2) and for the branches leading up to each of the four blattodean species (Table S3 – S5).

SUPPLEMENTARY FIGURES

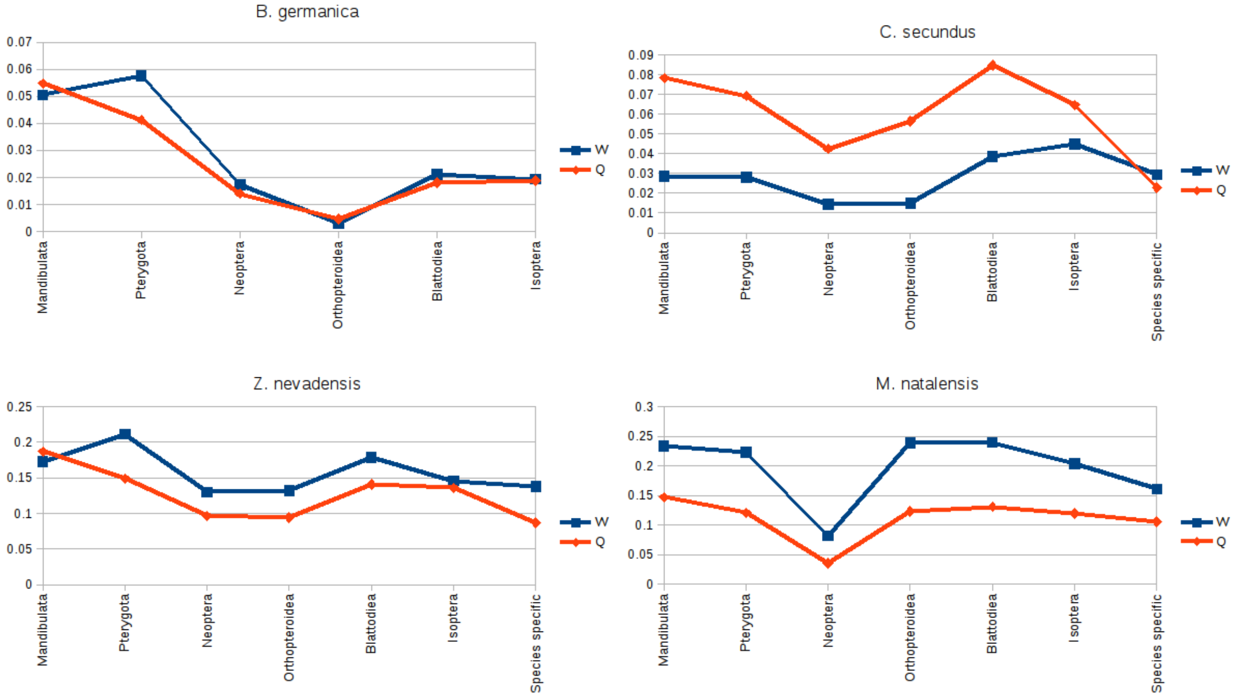

Figure S1: Proportions of differentially expressed genes according to gene age. Gene age or phylostratigraphy is represented along the x-axis and the proportion of genes which are worker-/nymph-biased (blue) or queen-/adult female-biased (orange) within each category are shown on the y-axis.

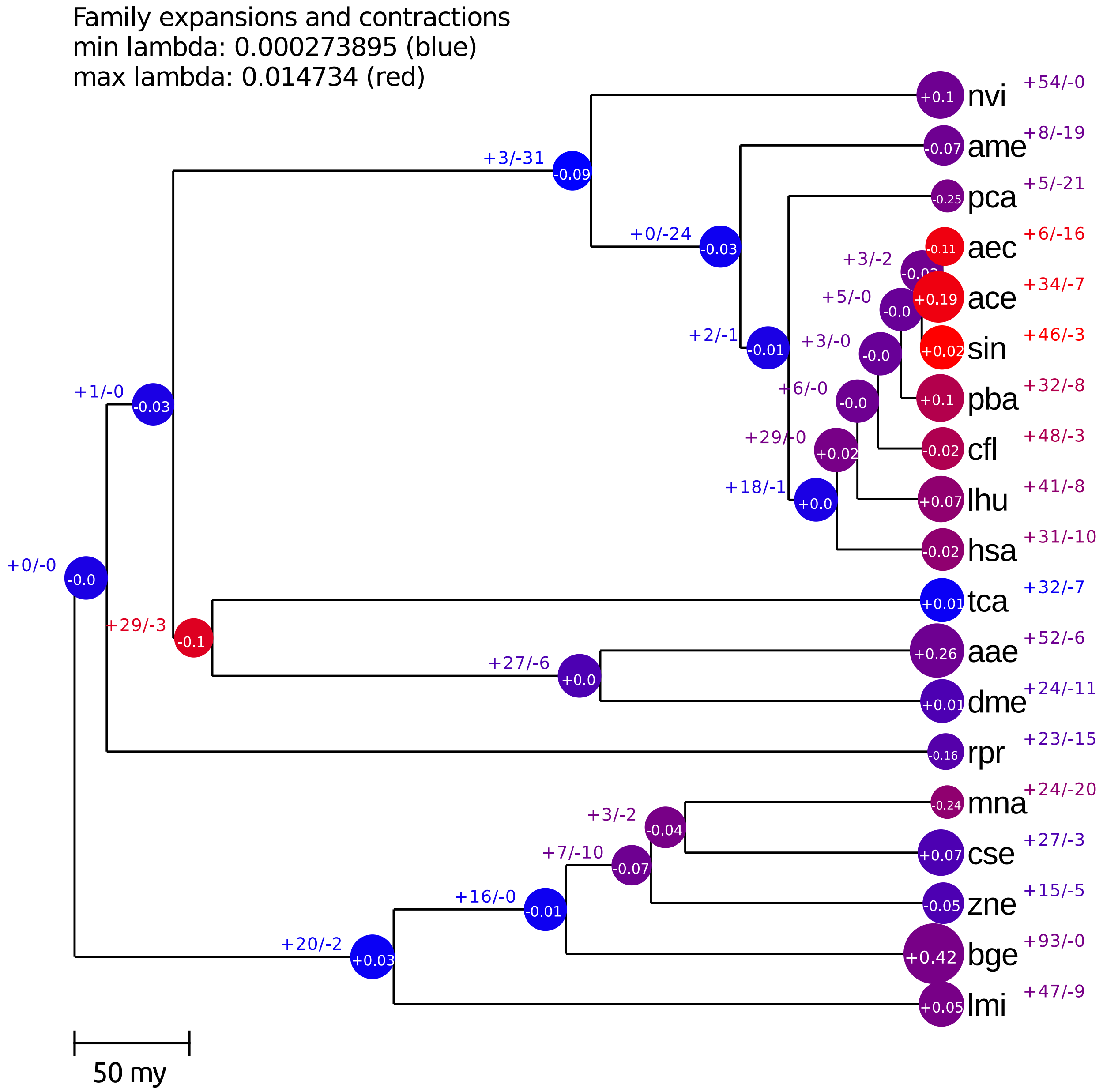

Figure S2: The number of significantly (Viterbi p-value < 0.01) expanded (+) and contracted (-) gene families across the phylogeny of 19 insects. The estimated rates of average gene gain and loss per gene per million years (λ) range from 0.00027 (blue) to 0.01473 (red). White decimal numbers indicate the mean number of genes gained or lost per family at that branch. nvi: *Nasonia vitripennis*; ame: *Apis mellifera*; aec: *Acromyrmex echinatior*; ace: *Atta cephalotes*; sin: *Solenopsis invicta*; pba: *Pogonomyrmex barbatus*; cfl: *Camponotus floridanus*; lhu: *Linepithema humile*; hsa: *Harpegnathos saltator*; tca: *Tribolium castaneum*; aae: *Aedes aegypti*; dme: *Drosophila melanogaster*; rpr: *Rhodnius prolixus*; mna: *Macrotermes natalensis*; cse: *Cryptotermes secundus*; zne: *Zootermopsis nevadensis*; bge: *Blattella germanica*; lmi: *Locusta migratoria*.

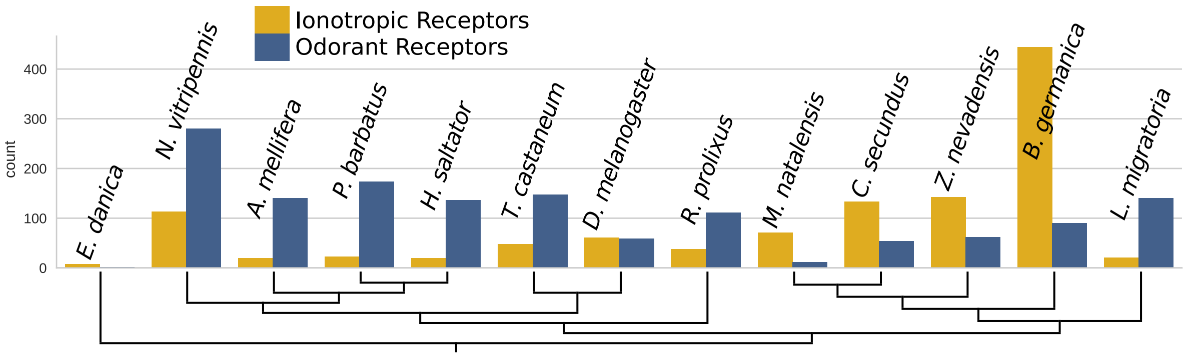

Figure S3: Counts of IRs (yellow) and ORs (blue) in 13 insect species. *B. germanica* counts are based on manual annotations, *Z. nevadensis* counts were previously published.[37] Other counts stem from HMM-based domain annotations of proteomes.

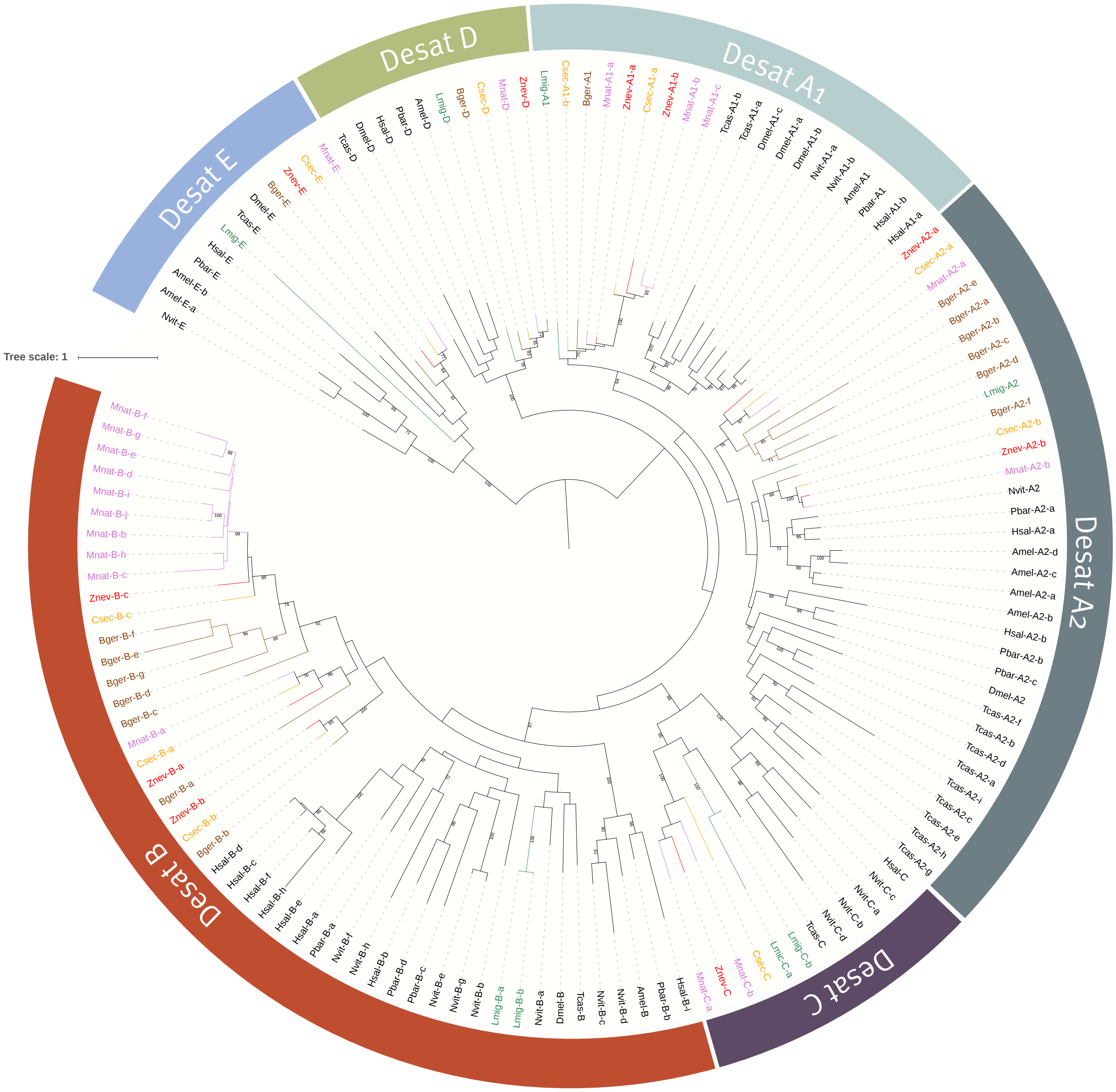

Figure S4: Gene tree of first desaturases. Tree is based on an untrimmed alignment due to very similar sequences. Only bootstrap values of 70 and above are shown.

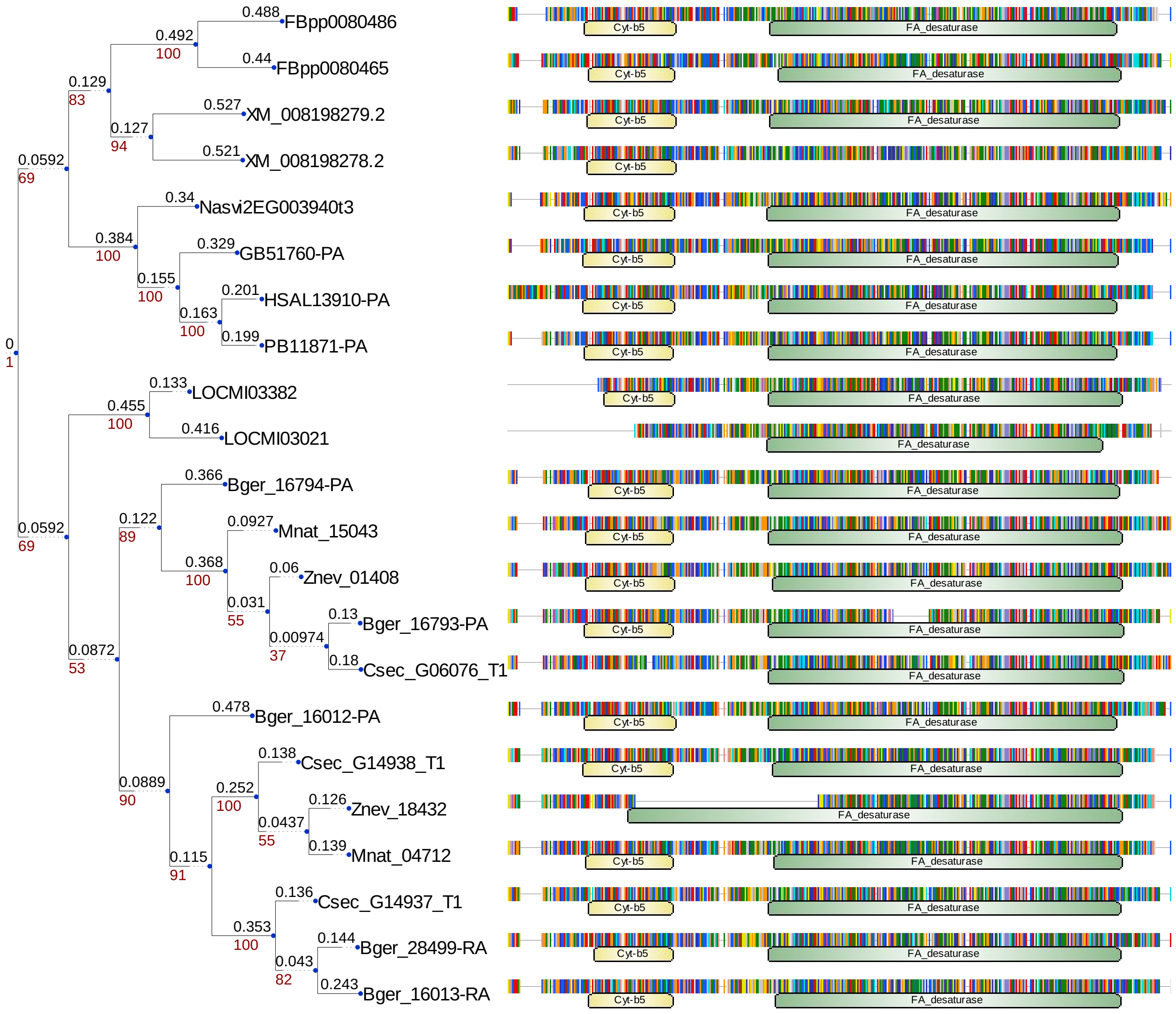

Figure S5: Gene tree of Desaturase-cytb5-r genes.

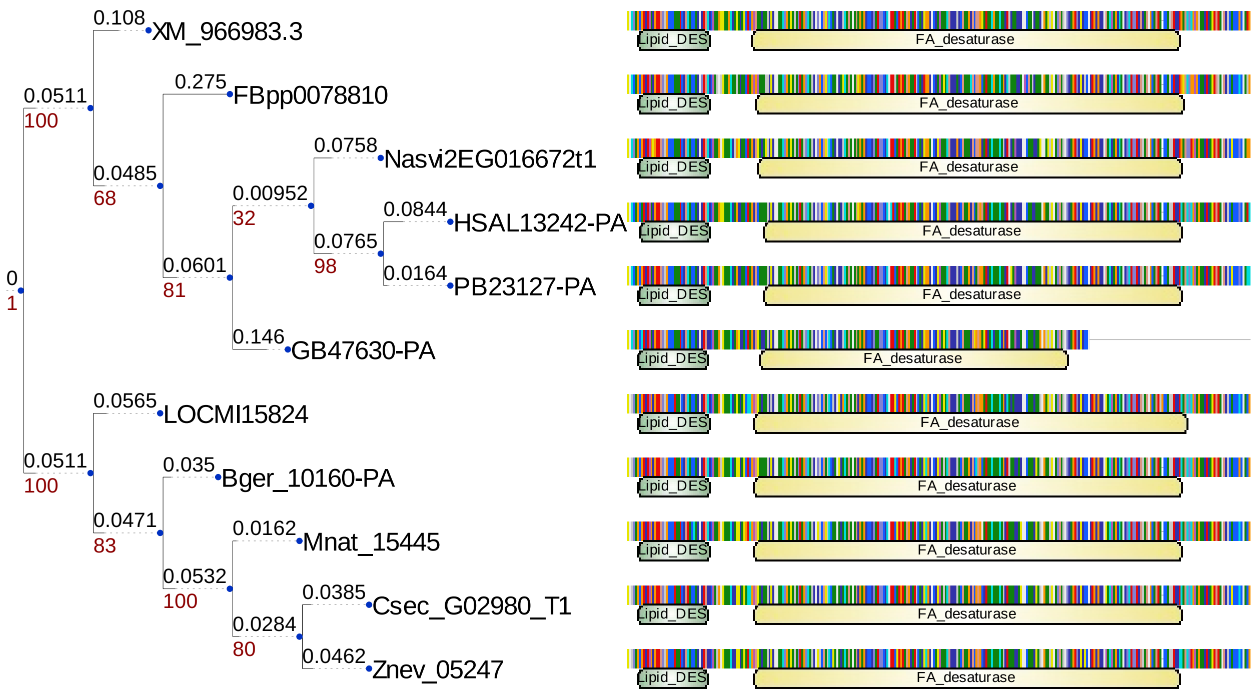

Figure S6: Analysis of the Ifc desaturases

SUPPLEMENTARY TABLES

Table S1: Genome assembly and annotation statistics for *B. germanica* and *C. secundus*, compared to the published genomes of *M. natalensis*, *Z. nevadensis* and *L. migratoria*. Statistics were generated with Quast (contiguity metrics), Busco2 (assembly completeness metrics) and Dogma2 (annotation completeness metrics).

Metric	*B. germanica*	*C. secundus*	*M. natalensis*	*Z. nevadensis*	*L. migratoria*
Estimated genome size (Mbp)	1 960	1 300	1 309	562	6 376
Total sequence length (bp)	2 037 201 033	1 019 133 518	1 172 292 920	493 468 737	6 905 712 912
Coverage	158x	45x	69x	98x	114x
No. scaffolds	24 820	55 493	145 794	93 931	564 328
No. contigs	317 391	89 222	268 397	127 321	1 403 616
Longest scaffold (bp)	7 469 083	6 593 022	10 840 804	5 111 804	8 179 469
Scaffold N50	1 056 071	1 184 893	2 016 407	756 883	333 431
Contig N50	12 126	61 785	17 767	22 357	10 002
Scaffold L50	576	245	168	190	3 512
Contig L50	39 323	4 744	17 024	6 147	167 858
GC (%)	34.33	41.07	39.84	38.19	40.67
No. N's per 100 kbp	16 041.78	2 055.09	4 948.74	4 446.76	15 987.71
Complete conserved single-copy orthologs (%)	79.74	81.79	85.0	90.32	50.73
Duplicated conserved single-copy orthologs (%)	1.12	0.97	0.56	0.60	1.57
Missing conserved single-copy orthologs (%)	4.90	4.07	3.44	1.91	20.15
Number of protein coding genes	29 216	18 162	16 140	15 459	17 363
Total domain completeness (%)	78.14	88.38	77.97	90.98	81.81
Single-domain completeness (%)	86.17	91.07	79.34	92.48	85.15

Table S2: Significant gene family contractions and expansions in the branch leading to Isoptera. "domain_content" denotes which domains occur in proteins of the cluster; the two numbers indicate how many of the genes in that cluster have the domain. "min|mean|max" refers to the family sizes of other (non-termite) insects. GO-terms are based on Pfam2GO.

cluster	domain content	size change	min|mean|max in other insects	domain description	domain GO-terms (Pfam2GO)
C_618272	Myb_DNA-bind_5 (89/112)	6 → 2 (p=0.0001)	0 | 6.6 | 39	Myb/SANT-like DNA-binding domain	-
C_616775	Chitin_bind_4 (411/418)	17 → 12 (p=0.0008)	11 | 23.3 | 67	Insect cuticle protein	GO:0042302 (structural constituent of cuticle)
C_606072	7tm_6 (53/53)	2 → 0 (p=0.0019)	0 | 3.3 | 47	7tm Odorant receptor	GO:0004984 (olfactory receptor activity); GO:0005549 (odorant binding); GO:0007608 (sensory perception of smell); GO:0016020 (membrane)
C_616310	Guanylate_cyc (22/41)	2 → 0 (p=0.0019)	0 | 2.6 | 9	Adenylate and Guanylate cyclase catalytic domain	GO:0009190 (cyclic nucleotide biosynthetic process); GO:0016849 (phosphorus-oxygen lyase activity); GO:0035556 (intracellular signal transduction)
C_615968	Pacifastin_I (29/29)	2 → 0 (p=0.0019)	0 | 1.8 | 13	Pacifastin inhibitor (LCMII)	GO:0030414 (peptidase inhibitor activity)
C_615899	CAP (169/185)	8 → 5 (p=0.0042)	1 | 9.8 | 26	Cysteine-rich secretory protein family	-
C_618396	EcKinase (533/539)	27 → 22 (p=0.0049)	12 | 29.1 | 54	Ecdysteroid kinase	-
C_596600	Trypsin (75/75)	3 → 1 (p=0.0052)	0 | 4.4 | 19	Trypsin	GO:0004252 (serine-type endopeptidase activity); GO:0006508 (proteolysis)
C_593628	Trypsin (481/481)	17 → 13 (p=0.0054)	6 | 27.5 | 78	Trypsin	GO:0004252 (serine-type endopeptidase activity); GO:0006508 (proteolysis)
C_618493	zf-H2C2_5 (64/454)	19 → 15 (p=0.0076)	0 | 26.2 | 48	C2H2-type zinc-finger domain	-
C_615399	Baculo_F (21/32)	1 → 5 (p=0.0000)	0 | 0.2 | 3	Baculovirus F protein	-
C_578794	zf-met (17/25)<br>zf-C2H2 (17/25)	1 → 5 (p=0.0000)	0 | 0.1 | 1	Zinc-finger of C2H2 type<br>Zinc finger, C2H2 type	-<br>GO:0046872 (metal ion binding)
Ir_group_B		21 → 30 (p=0.0000)	0 | 5.9 | 36		
C_532862	zf-C2H2 (63/81)<br>zf-met (54/81)<br>zf-C2H2_4 (38/81)<br>zf-C2H2_6 (35/81)	10 → 17 (p=0.0000)	0 | 1.1 | 13	Zinc finger, C2H2 type<br>Zinc-finger of C2H2 type<br>C2H2-type zinc finger<br>C2H2-type zinc finger	GO:0046872 (metal ion binding)<br>-<br>-<br>GO:0046872 (metal ion binding)
C_617527	MADF_DNA_bdg (36/47)<br>BESS (29/47)	3 → 6 (p=0.0008)	0 | 1.4 | 4	Alcohol dehydrogenase transcription factor Myb/SANT-like<br>BESS motif	-<br>GO:0003677 (DNA binding)

C_538517	zf-C2H2_6 (46/70)	7 → 10 (p=0.0077)	0 | 1.9 | 15	C2H2-type zinc finger	GO:0046872 (metal ion binding)
	zf-C2H2 (41/70)			Zinc finger, C2H2 type	GO:0046872 (metal ion binding)
	zf-met (40/70)			Zinc-finger of C2H2 type	-
	zf-C2H2_4 (31/70)			C2H2-type zinc finger	-
C_586207	zf-C2H2 (188/376)	27 → 32 (p=0.0096)	0 | 15.4 | 39	Zinc finger, C2H2 type	GO:0046872 (metal ion binding)
	zf-met (143/376)			Zinc-finger of C2H2 type	-
	zf-C2H2_6 (135/376)			C2H2-type zinc finger	GO:0046872 (metal ion binding)
	zf-C2H2_4 (113/376)			C2H2-type zinc finger	-

Table S3: Significant gene family contractions and expansions in *Zootermopsis nevadensis*. "domain_content" denotes which domains occur in proteins of the cluster; the two numbers indicate how many of the genes in that cluster have the domain. "min|mean|max" refers to the family sizes of other insects. GO-terms are based on Pfam2GO.

cluster	domain content	size change	min|mean|max in other insects	domain description	domain GO-terms (Pfam2GO)
C_618161	Dynein_heavy (306/474)<br>AAA_9 (268/474)<br>MT (267/474)<br>AAA_6 (259/474)<br>AAA_8 (256/474)<br>DHC_N2 (255/474)<br>AAA_7 (236/474)<br>DHC_N1 (154/474)<br>AAA_5 (147/474)	19 → 8 (p=0.0000)	8 | 22.6 | 58	Dynein heavy chain and region D6 of dynein motor<br>ATP-binding dynein motor region D5<br>Microtubule-binding stalk of dynein motor<br>Hydrolytic ATP binding site of dynein motor region D1<br>P-loop containing dynein motor region D4<br>Dynein heavy chain, N-terminal region 2<br>P-loop containing dynein motor region D3<br>Dynein heavy chain, N-terminal region 1<br>AAA domain (dynein-related subfamily)	GO:0003777 (microtubule motor activity); GO:0007018 (microtubule-based movement); GO:0030286 (dynein complex)<br>-<br>-<br>-<br>-<br>-<br>-<br>-<br>GO:0005524 (ATP binding); GO:0016887 (ATPase activity)
C_592862	p450 (686/687)	31 → 18 (p=0.0000)	10 | 34.7 | 91	Cytochrome P450	GO:0005506 (iron ion binding); GO:0016705 (oxidoreductase activity, acting on paired donors, with incorporation or reduction of molecular oxygen); GO:0020037 (heme binding); GO:0055114 (oxidation-reduction process)
C_616288	ketoacyl-synt (202/384)<br>Ketoacyl-synt_C (171/384)<br>Acyl_transf_1 (132/384)<br>KAsynt_C_assoc (126/384)<br>KR (109/384)<br>PS-DH (98/384)	13 → 8 (p=0.0058)	3 | 19.1 | 100	Beta-ketoacyl synthase, N-terminal domain<br>Beta-ketoacyl synthase, C-terminal domain<br>Acyl transferase domain<br>Ketoacyl-synthetase C-terminal extension<br>KR domain<br>Polyketide synthase dehydratase	-<br>-<br>-<br>-<br>-<br>-
C_615899	CAP (169/185)	5 → 2 (p=0.0092)	1 | 8.7 | 26	Cysteine-rich secretory protein family	-
C_618445	Acyl_transf_3 (245/365)	15 → 10 (p=0.0094)	6 | 16.3 | 48	Acyltransferase family	GO:0016747 (transferase activity, transferring acyl groups other than amino-acyl groups)
C_612885	F-box-like (9/34)	1 → 21 (p=0.0000)	0 | 1.8 | 21	F-box-like	GO:0005515 (protein binding)
C_612931	7tm_7 (63/70)	9 → 24 (p=0.0000)	0 | 3.7 | 27	7tm Chemosensory receptor	GO:0016021 (integral component of membrane); GO:0050909 (sensory perception of taste)
C_617309	Kelch_1 (7/21)	1 → 14 (p=0.0000)	0 | 1.1 | 14	Kelch motif	GO:0005515 (protein binding)
C_612340		1 → 14 (p=0.0000)	0 | 0.9 | 14		
C_613520	PKD_channel (30/31)	2 → 9 (p=0.0000)	0 | 1.5 | 9	Polycystin cation channel	-

C_618577	RYDR_ITPR (52/77)<br>RIH_assoc (41/77)<br>Ins145_P3_rec (41/77)<br>Ion_trans (40/77)<br>MIR (39/77)<br>RyR (29/77)<br>SPRY (21/77)	4 $\rightarrow$ 11 (p=0.0000)	2 | 3.5 | 11	RIH domain<br>RyR and IP3R Homology associated<br>Inositol 1,4,5-trisphosphate/ryanodine receptor<br>Ion transport protein<br>MIR domain<br>RyR domain<br>SPRY domain	GO:0005262 (calcium channel activity); GO:0016020 (membrane); GO:0070588 (calcium ion transmembrane transport)<br>-<br>-<br>GO:0005216 (ion channel activity); GO:0006811 (ion transport); GO:0016020 (membrane); GO:0055085 (transmembrane transport)<br>GO:0016020 (membrane)<br>-<br>GO:0005515 (protein binding)
Ir_group_B		30 $\rightarrow$ 44 (p=0.0000)	0 | 10.4 | 44		
C_616701	Pkinase (19/45)<br>Pkinase_C (15/45)	3 $\rightarrow$ 9 (p=0.0000)	0 | 2.2 | 9	Protein kinase domain<br>Protein kinase C terminal domain	GO:0004672 (protein kinase activity); GO:0005524 (ATP binding); GO:0006468 (protein phosphorylation)<br>GO:0004674 (protein serine/threonine kinase activity); GO:0005524 (ATP binding); GO:0006468 (protein phosphorylation)
C_591836	7tm_7 (79/79)	3 $\rightarrow$ 9 (p=0.0000)	0 | 4.2 | 56	7tm Chemosensory receptor	GO:0016021 (integral component of membrane); GO:0050909 (sensory perception of taste)
Ir_group_A		11 $\rightarrow$ 20 (p=0.0000)	3 | 9.5 | 20		
C_532862	zf-C2H2 (63/81)<br>zf-met (54/81)<br>zf-C2H2_4 (38/81)<br>zf-C2H2_6 (35/81)	17 $\rightarrow$ 27 (p=0.0000)	0 | 4.2 | 27	Zinc finger, C2H2 type<br>Zinc-finger of C2H2 type<br>C2H2-type zinc finger<br>C2H2-type zinc finger	GO:0046872 (metal ion binding)<br>-<br>-<br>GO:0046872 (metal ion binding)
C_592648	Pkinase (576/577)<br>Pkinase_C (152/577)	33 $\rightarrow$ 45 (p=0.0002)	18 | 27.8 | 45	Protein kinase domain<br>Protein kinase C terminal domain	GO:0004672 (protein kinase activity); GO:0005524 (ATP binding); GO:0006468 (protein phosphorylation)<br>GO:0004674 (protein serine/threonine kinase activity); GO:0005524 (ATP binding); GO:0006468 (protein phosphorylation)
C_618160	HMG_box (264/307)	16 $\rightarrow$ 24 (p=0.0007)	8 | 14.3 | 24	HMG (high mobility group) box	-
C_605543		1 $\rightarrow$ 3 (p=0.0061)	0 | 0.7 | 4		
C_616813	LRR_6 (121/221)<br>F-box-like (111/221)	11 $\rightarrow$ 16 (p=0.0068)	6 | 10.6 | 21	Leucine Rich repeat<br>F-box-like	GO:0005515 (protein binding)<br>GO:0005515 (protein binding)

Table S4: Significant gene family contractions and expansions in *Cryptotermes secundus*. "domain_content" denotes which domains occur in proteins of the cluster; the two numbers indicate how many of the genes in that cluster have the domain. "min|mean|max" refers to the family sizes of other insects. GO-terms are based on Pfam2GO.

cluster	domain content	size change	min|mean|max in other insects	domain description	domain GO-terms (Pfam2GO)
C_618279	-	9 → 1 (p=0.0000)	0 | 9.7 | 69	-	-
C_618183	-	9 → 5 (p=0.0058)	0 | 10.4 | 66	-	-
C_613289	p450 (83/91)	2 → 0 (p=0.0080)	0 | 4.1 | 15	Cytochrome P450	GO:0005506 (iron ion binding); GO:0016705 (oxidoreductase activity, acting on paired donors, with incorporation or reduction of molecular oxygen); GO:0020037 (heme binding); GO:0055114 (oxidation-reduction process)
C_617666		5 → 37 (p=0.0000)	0 | 2.3 | 37		
C_608697	-	16 → 36 (p=0.0000)	0 | 5.3 | 43	-	-
C_615449	Sina (232/301)	37 → 57 (p=0.0000)	3 | 15.5 | 90	Seven in absentia protein family	GO:0005634 (nucleus); GO:0006511 (ubiquitin-dependent protein catabolic process); GO:0007275 (multicellular organism development)
C_617923	VPS13 (76/130)<br>SHR-BD (72/130)<br>VPS13_C (70/130)<br>Chorein_N (70/130)<br>VPS13_mid_rpt (55/130)	7 → 19 (p=0.0000)	3 | 6.1 | 19	Vacuolar sorting-associated protein 13, N-terminal<br>SHR-binding domain of vacuolar-sorting associated protein 13<br>Vacuolar-sorting-associated 13 protein C-terminal<br>N-terminal region of Chorein or VPS13<br>Repeating coiled region of VPS13	-<br>-<br>-<br>-<br>-
C_617306	MADF_DNA_bdg (68/80)<br>BESS (68/80)	6 → 16 (p=0.0000)	1 | 4.2 | 16	Alcohol dehydrogenase transcription factor Myb/SANT-like<br>BESS motif	-<br>GO:0003677 (DNA binding)
C_615399	Baculo_F (21/32)	6 → 23 (p=0.0000)	0 | 1.7 | 23	Baculovirus F protein	-
C_611352	Kin17_mid (33/42)	2 → 14 (p=0.0000)	1 | 2.1 | 14	Domain of Kin17 curved DNA-binding protein	-
C_604491	B56 (53/54)	3 → 12 (p=0.0000)	1 | 2.6 | 12	Protein phosphatase 2A regulatory B subunit (B56 family)	GO:0000159 (protein phosphatase type 2A complex); GO:0007165 (signal transduction)

C_581761	zf-C2H2_6 (359/525)<br>zf-met (310/525)<br>zf-C2H2 (303/525)<br>zf-C2H2_4 (244/525)<br>zf-AD (157/525)	52 → 97 (p=0.0000)	1 | 26.5 | 97	C2H2-type zinc finger<br>Zinc-finger of C2H2 type<br>Zinc finger, C2H2 type<br>C2H2-type zinc finger<br>Zinc-finger associated domain (zf-AD)	GO:0046872 (metal ion binding)<br>-<br>GO:0046872 (metal ion binding)<br>-<br>GO:0005634 (nucleus); GO:0008270 (zinc ion binding)
C_617527	MADF_DNA_bdg (36/47)<br>BESS (29/47)	6 → 16 (p=0.0000)	0 | 2.5 | 16	Alcohol dehydrogenase transcription factor Myb/SANT-like<br>BESS motif	-<br>GO:0003677 (DNA binding)
C_615780	MADF_DNA_bdg (90/107)	5 → 13 (p=0.0000)	0 | 5.6 | 73	Alcohol dehydrogenase transcription factor Myb/SANT-like	-
C_618464	MADF_DNA_bdg (71/81)	6 → 13 (p=0.0000)	0 | 4.2 | 20	Alcohol dehydrogenase transcription factor Myb/SANT-like	-
C_618053	CTD_bind (18/37)<br>CFIA_Pcf11 (12/37)	2 → 7 (p=0.0000)	0 | 1.8 | 7	RNA polymerase II-binding domain.<br>Subunit of cleavage factor IA Pcf11	-<br>GO:0005849 (mRNA cleavage factor complex); GO:0006369 (termination of RNA polymerase II transcription); GO:0006378 (mRNA polyadenylation); GO:0006379 (mRNA cleavage)
C_615705	F-box-like (47/62)	8 → 15 (p=0.0001)	0 | 3.2 | 17	F-box-like	GO:0005515 (protein binding)
C_615752	Tubulin (320/343)<br>Tubulin_C (301/343)	26 → 37 (p=0.0001)	11 | 16.8 | 37	Tubulin/FtsZ family, GTPase domain<br>Tubulin C-terminal domain	GO:0003924 (GTPase activity)<br>-
C_608483	7tm_6 (44/47)	1 → 4 (p=0.0005)	1 | 2.3 | 21	7tm Odorant receptor	GO:0004984 (olfactory receptor activity); GO:0005549 (odorant binding); GO:0007608 (sensory perception of smell); GO:0016020 (membrane)
C_618161	Dynein_heavy (306/474)<br>AAA_9 (268/474)<br>MT (267/474)<br>AAA_6 (259/474)<br>AAA_8 (256/474)<br>DHC_N2 (255/474)<br>AAA_7 (236/474)<br>DHC_N1 (154/474)<br>AAA_5 (147/474)	19 → 27 (p=0.0005)	8 | 22.6 | 58	Dynein heavy chain and region D6 of dynein motor<br>ATP-binding dynein motor region D5<br>Microtubule-binding stalk of dynein motor<br>Hydrolytic ATP binding site of dynein motor region D1<br>P-loop containing dynein motor region D4<br>Dynein heavy chain, N-terminal region 2<br>P-loop containing dynein motor region D3<br>Dynein heavy chain, N-terminal region 1<br>AAA domain (dynein-related subfamily)	GO:0003777 (microtubule motor activity); GO:0007018 (microtubule-based movement); GO:0030286 (dynein complex)<br>-<br>-<br>-<br>-<br>-<br>-<br>-<br>GO:0005524 (ATP binding); GO:0016887 (ATPase activity)
C_578794	zf-met (17/25)<br>zf-C2H2 (17/25)	6 → 11 (p=0.0006)	0 | 1.3 | 11	Zinc-finger of C2H2 type<br>Zinc finger, C2H2 type	-<br>GO:0046872 (metal ion binding)

C_617871	MADF_DNA_bdg (89/98)<br>BESS (45/98)	6 → 11 (p=0.0006)	0 | 4.8 | 13	Alcohol dehydrogenase transcription factor Myb/SANT-like<br>BESS motif	-<br>GO:0003677 (DNA binding)
C_612313	Endonuclease_NS (81/87)	4 → 8 (p=0.0012)	0 | 4.3 | 23	DNA/RNA non-specific endonuclease	GO:0003676 (nucleic acid binding); GO:0016787 (hydrolase activity); GO:0046872 (metal ion binding)
C_532862	zf-C2H2 (63/81)<br>zf-met (54/81)<br>zf-C2H2_4 (38/81)<br>zf-C2H2_6 (35/81)	17 → 24 (p=0.0012)	0 | 4.2 | 27	Zinc finger, C2H2 type<br>Zinc-finger of C2H2 type<br>C2H2-type zinc finger<br>C2H2-type zinc finger	GO:0046872 (metal ion binding)<br>-<br>-<br>GO:0046872 (metal ion binding)
C_592784	p450 (52/54)	3 → 6 (p=0.0046)	0 | 2.8 | 13	Cytochrome P450	GO:0005506 (iron ion binding); GO:0016705 (oxidoreductase activity, acting on paired donors, with incorporation or reduction of molecular oxygen); GO:0020037 (heme binding); GO:0055114 (oxidation-reduction process)
C_538517	zf-C2H2_6 (46/70)<br>zf-C2H2 (41/70)<br>zf-met (40/70)<br>zf-C2H2_4 (31/70)	11 → 16 (p=0.0046)	0 | 3.6 | 16	C2H2-type zinc finger<br>Zinc finger, C2H2 type<br>Zinc-finger of C2H2 type<br>C2H2-type zinc finger	GO:0046872 (metal ion binding)<br>GO:0046872 (metal ion binding)<br>-<br>-
C_615823		1 → 3 (p=0.0049)	0 | 0.3 | 3		
C_616311	Cnd1_N (20/41)<br>Cnd1 (20/41)	1 → 3 (p=0.0049)	0 | 2.1 | 17	non-SMC mitotic condensation complex subunit 1, N-term<br>non-SMC mitotic condensation complex subunit 1	-<br>-
C_606925	Ig_3 (20/32)<br>I-set (12/32)	1 → 3 (p=0.0049)	0 | 1.4 | 3	Immunoglobulin domain<br>Immunoglobulin I-set domain	-<br>-
C_617307	MADF_DNA_bdg (9/15)<br>BESS (5/15)	1 → 3 (p=0.0049)	0 | 0.8 | 3	Alcohol dehydrogenase transcription factor Myb/SANT-like<br>BESS motif	-<br>GO:0003677 (DNA binding)

Table S5: Significant gene family contractions and expansions in *Macrotermes natalensis*. "domain_content" denotes which domains occur in proteins of the cluster; the two numbers indicate how many of the genes in that cluster have the domain. "min|mean|max" refers to the family sizes of other insects. GO-terms are based on Pfam2GO.

cluster	domain content	size change	min|mean|max in other insects	domain description	domain GO-terms (Pfam2GO)
C_607839	7tm_6 (385/388)	24 → 3 (p=0.0000)	1 | 20.4 | 86	7tm Odorant receptor	GO:0004984 (olfactory receptor activity); GO:0005549 (odorant binding); GO:0007608 (sensory perception of smell); GO:0016020 (membrane)
C_581761	zf-C2H2_6 (359/525)<br>zf-met (310/525)<br>zf-C2H2 (303/525)<br>zf-C2H2_4 (244/525)<br>zf-AD (157/525)	52 → 25 (p=0.0000)	1 | 26.5 | 97	C2H2-type zinc finger<br>Zinc-finger of C2H2 type<br>Zinc finger, C2H2 type<br>C2H2-type zinc finger<br>Zinc-finger associated domain (zf-AD)	GO:0046872 (metal ion binding)<br>-<br>GO:0046872 (metal ion binding)<br>-<br>GO:0005634 (nucleus); GO:0008270 (zinc ion binding)
C_618287	Lectin_C (163/169)	13 → 2 (p=0.0000)	0 | 8.8 | 86	Lectin C-type domain	-
C_608697	-	16 → 4 (p=0.0000)	0 | 5.3 | 43	-	-
C_607490	COesterase (713/713)	35 → 17 (p=0.0000)	14 | 34.7 | 102	Carboxylesterase family	-
C_593628	Trypsin (481/481)	12 → 2 (p=0.0000)	2 | 24.5 | 78	Trypsin	GO:0004252 (serine-type endopeptidase activity); GO:0006508 (proteolysis)
C_615449	Sina (232/301)	37 → 20 (p=0.0001)	3 | 15.5 | 90	Seven in absentia protein family	GO:0005634 (nucleus); GO:0006511 (ubiquitin-dependent protein catabolic process); GO:0007275 (multicellular organism development)
C_617921	-	17 → 6 (p=0.0002)	6 | 13.8 | 35	-	-
C_617527	MADF_DNA_bdg (36/47)<br>BESS (29/47)	6 → 0 (p=0.0002)	0 | 2.5 | 16	Alcohol dehydrogenase transcription factor Myb/SANT-like<br>BESS motif	-<br>GO:0003677 (DNA binding)
C_615738	HLH (392/398)	18 → 7 (p=0.0002)	3 | 18.5 | 27	Helix-loop-helix DNA-binding domain	GO:0046983 (protein dimerization activity)
C_615780	MADF_DNA_bdg (90/107)	5 → 0 (p=0.0010)	0 | 5.6 | 73	Alcohol dehydrogenase transcription factor Myb/SANT-like	-
C_615705	F-box-like (47/62)	8 → 2 (p=0.0037)	0 | 3.2 | 17	F-box-like	GO:0005515 (protein binding)
C_604319	Chitin_bind_4 (135/139)	8 → 2 (p=0.0037)	2 | 6.8 | 20	Insect cuticle protein	GO:0042302 (structural constituent of cuticle)
C_615752	Tubulin (320/343)<br>Tubulin_C (301/343)	26 → 15 (p=0.0041)	11 | 16.8 | 37	Tubulin/FtsZ family, GTPase domain<br>Tubulin C-terminal domain	GO:0003924 (GTPase activity)<br>-
C_615399	Baculo_F (21/32)	6 → 1 (p=0.0046)	0 | 1.7 | 23	Baculovirus F protein	-

C_603425	Trypsin (145/145)<br>GD_N (39/145)	4 → 0 (p=0.0048)	0 | 6.9 | 37	Trypsin<br>Serine protease gd N-terminus	GO:0004252 (serine-type endopeptidase activity); GO:0006508 (proteolysis)<br>-
C_612931	7tm_7 (63/70)	9 → 3 (p=0.0058)	0 | 3.7 | 27	7tm Chemosensory receptor	GO:0016021 (integral component of membrane); GO:0050909 (sensory perception of taste)
C_618160	HMG_box (264/307)	16 → 8 (p=0.0066)	8 | 14.3 | 24	HMG (high mobility group) box	-
Ir_group_C		7 → 2 (p=0.0081)	1 | 5.2 | 23		
C_612521	Lipase (442/443)	17 → 9 (p=0.0083)	9 | 21.7 | 46	Lipase	-
C_618426		4 → 40 (p=0.0000)	0 | 2.4 | 40		
C_596573	LRR_8 (145/149)<br>I-set (55/149)<br>Ig_3 (40/149)	9 → 26 (p=0.0000)	4 | 7.1 | 26	Leucine rich repeat<br>Immunoglobulin I-set domain<br>Immunoglobulin domain	GO:0005515 (protein binding)<br>-<br>-
C_614083	Proton_antipo_M (45/50)	2 → 14 (p=0.0000)	0 | 2.3 | 14	Proton-conducting membrane transporter	-
C_614079	7tm_1 (132/144)	16 → 45 (p=0.0000)	0 | 7.5 | 45	7 transmembrane receptor (rhodopsin family)	GO:0004930 (G-protein coupled receptor activity); GO:0007186 (G-protein coupled receptor signaling pathway); GO:0016021 (integral component of membrane)
C_615611	BIR (161/207)<br>zf-C3HC4_3 (101/207)	8 → 28 (p=0.0000)	3 | 8.3 | 28	Inhibitor of Apoptosis domain<br>Zinc finger, C3HC4 type (RING finger)	-<br>-
C_618279	-	9 → 69 (p=0.0000)	0 | 9.7 | 69	-	-
C_618183	-	9 → 66 (p=0.0000)	0 | 10.4 | 66	-	-
C_616091	Proton_antipo_M (65/76)	3 → 15 (p=0.0000)	0 | 3.5 | 17	Proton-conducting membrane transporter	-
C_614954		1 → 10 (p=0.0000)	0 | 0.6 | 10		
C_618433	C_tripleX (2/69)	1 → 9 (p=0.0000)	0 | 3.6 | 49	Cysteine rich repeat	-
C_615859	Cytochrome_B (10/27)<br>Cytochrom_B_N_2 (7/27)	1 → 8 (p=0.0000)	0 | 1.3 | 8	Cytochrome b/b6/petB<br>Cytochrome b(N-terminal)/b6/petB	GO:0009055 (electron carrier activity); GO:0016020 (membrane); GO:0016491 (oxidoreductase activity)<br>GO:0009055 (electron carrier activity); GO:0016020 (membrane); GO:0016491 (oxidoreductase activity)
C_615489	NADHdh (46/46)	2 → 10 (p=0.0000)	0 | 2.2 | 10	NADH dehydrogenase	GO:0016020 (membrane); GO:0055114 (oxidation-reduction process)
C_618775	-	5 → 15 (p=0.0000)	0 | 3.5 | 27	-	-
C_617231		1 → 7 (p=0.0001)	0 | 0.5 | 7		
desat_B		3 → 10 (p=0.0003)	1 | 4.3 | 10		

C_616610	-	1 → 5 (p=0.0016)	0 | 0.4 | 5	-	-
C_616288	ketoacyl-synt (202/384)	15 → 26 (p=0.0019)	3 | 19.1 | 100	Beta-ketoacyl synthase, N-terminal domain	-
	Ketoacyl-synt_C (171/384)			Beta-ketoacyl synthase, C-terminal domain	-
	Acyl_transf_1 (132/384)			Acyl transferase domain	-
	KAsynt_C_assoc (126/384)			Ketoacyl-synthetase C-terminal extension	-
	KR (109/384)			KR domain	-
	PS-DH (98/384)			Polyketide synthase dehydratase	-
C_613978	FA_desaturase (193/253)	12 → 21 (p=0.0033)	7 | 12.8 | 29	Fatty acid desaturase	GO:0006629 (lipid metabolic process)
C_616527	C2-set_2 (11/34)	1 → 4 (p=0.0065)	0 | 1.7 | 4	CD80-like C2-set immunoglobulin domain	-
C_618088		1 → 4 (p=0.0065)	0 | 0.3 | 4		
C_616975		1 → 4 (p=0.0065)	0 | 0.3 | 4		
C_611864	Acyl_transf_1 (6/18)	1 → 4 (p=0.0065)	0 | 0.9 | 11	Acyl transferase domain	-
C_618258	PT (22/23)	1 → 4 (p=0.0065)	0 | 1.2 | 15	PT repeat	-
C_618273	ANF_receptor (24/35)	1 → 4 (p=0.0065)	0 | 1.7 | 5	Receptor family ligand binding region	-

Table S6: Significant gene family contractions and expansions in *Blattella germanica*. "domain_content" denotes which domains occur in proteins of the cluster; the two numbers indicate how many of the genes in that cluster have the domain. "min|mean|max" refers to the family sizes of other insects. GO-terms are based on Pfam2GO.

cluster	domain content	size change	min|mean|max in other insects	domain description	domain GO-terms (Pfam2GO)
C_607839	7tm_6 (385/388)	32 → 74 (p=0.0000)	1 | 20.4 | 86	7tm Odorant receptor	GO:0004984 (olfactory receptor activity); GO:0005549 (odorant binding); GO:0007608 (sensory perception of smell); GO:0016020 (membrane)
C_596427	Sugar_tr (661/661)	41 → 74 (p=0.0000)	19 | 33.7 | 74	Sugar (and other) transporter	GO:0016021 (integral component of membrane); GO:0022857 (transmembrane transporter activity); GO:0055085 (transmembrane transport)
C_618445	Acyl_transf_3 (245/365)	16 → 48 (p=0.0000)	6 | 16.3 | 48	Acyltransferase family	GO:0016747 (transferase activity, transferring acyl groups other than amino-acyl groups)
C_613988	Transmemb_17 (23/27)	1 → 16 (p=0.0000)	0 | 1.4 | 16	Predicted membrane protein	-
C_564414	zf-C2H2_4 (39/56)<br>zf-AD (35/56)<br>zf-C2H2_6 (34/56)<br>zf-C2H2 (25/56)<br>zf-met (17/56)	3 → 17 (p=0.0000)	0 | 2.9 | 17	C2H2-type zinc finger<br>Zinc-finger associated domain (zf-AD)<br>C2H2-type zinc finger<br>Zinc finger, C2H2 type<br>Zinc-finger of C2H2 type	-<br>GO:0005634 (nucleus); GO:0008270 (zinc ion binding)<br>GO:0046872 (metal ion binding)<br>GO:0046872 (metal ion binding)<br>-
C_618767	F-box-like (23/47)<br>F-box (13/47)	1 → 21 (p=0.0000)	0 | 2.5 | 21	F-box-like<br>F-box domain	GO:0005515 (protein binding)<br>GO:0005515 (protein binding)
C_608697	-	13 → 43 (p=0.0000)	0 | 5.3 | 43	-	-
C_615449	Sina (232/301)	31 → 90 (p=0.0000)	3 | 15.5 | 90	Seven in absentia protein family	GO:0005634 (nucleus); GO:0006511 (ubiquitin-dependent protein catabolic process); GO:0007275 (multicellular organism development)
C_596597	Ank_2 (47/55)<br>NACHT (26/55)<br>Ank (26/55)<br>Ank_4 (18/55)	5 → 28 (p=0.0000)	0 | 2.9 | 28	Ankyrin repeats (3 copies)<br>NACHT domain<br>Ankyrin repeat<br>Ankyrin repeats (many copies)	-<br>-<br>GO:0005515 (protein binding)<br>-
C_614079	7tm_1 (132/144)	13 → 40 (p=0.0000)	0 | 7.5 | 45	7 transmembrane receptor (rhodopsin family)	GO:0004930 (G-protein coupled receptor activity); GO:0007186 (G-protein coupled receptor signaling pathway); GO:0016021 (integral component of membrane)
C_589090	7tm_7 (65/65)	3 → 55 (p=0.0000)	0 | 3.4 | 55	7tm Chemosensory receptor	GO:0016021 (integral component of membrane); GO:0050909 (sensory perception of taste)
C_612931	7tm_7 (63/70)	8 → 27 (p=0.0000)	0 | 3.7 | 27	7tm Chemosensory receptor	GO:0016021 (integral component of membrane); GO:0050909 (sensory perception of taste)

C_618470	-	5 → 40 (p=0.0000)	0 | 6.5 | 51	-	-
C_613764	UDPGT (515/542)	30 → 61 (p=0.0000)	9 | 26.9 | 72	UDP-glucoronosyl and UDP-glucosyl transferase	GO:0008152 (metabolic process); GO:0016758 (transferase activity, transferring hexosyl groups)
C_618287	Lectin_C (163/169)	14 → 86 (p=0.0000)	0 | 8.8 | 86	Lectin C-type domain	-
Ir_group_C		7 → 23 (p=0.0000)	1 | 5.2 | 23		
C_616112	-	1 → 11 (p=0.0000)	0 | 1.6 | 11	-	-
C_618775	-	5 → 27 (p=0.0000)	0 | 3.5 | 27	-	-
C_617624	Acetyltransf_1 (58/186)	9 → 26 (p=0.0000)	5 | 9.3 | 26	Acetyltransferase (GNAT) family	GO:0008080 (N-acetyltransferase activity)
C_612874	zf-AD (22/23)	2 → 13 (p=0.0000)	0 | 1.2 | 13	Zinc-finger associated domain (zf-AD)	GO:0005634 (nucleus); GO:0008270 (zinc ion binding)
C_614250	GMC_oxred_N (492/526)<br>GMC_oxred_C (471/526)	29 → 64 (p=0.0000)	14 | 26.3 | 64	GMC oxidoreductase<br>GMC oxidoreductase	GO:0016614 (oxidoreductase activity, acting on CH-OH group of donors); GO:0050660 (flavin adenine dinucleotide binding); GO:0055114 (oxidation-reduction process)<br>GO:0016614 (oxidoreductase activity, acting on CH-OH group of donors); GO:0055114 (oxidation-reduction process)
C_615733	ASC (301/302)	16 → 42 (p=0.0000)	6 | 14.9 | 42	Amiloride-sensitive sodium channel	GO:0005272 (sodium channel activity); GO:0006814 (sodium ion transport); GO:0016020 (membrane)
C_618279	-	9 → 51 (p=0.0000)	0 | 9.7 | 69	-	-
C_618272	Myb_DNA-bind_5 (89/112)	6 → 39 (p=0.0000)	0 | 5.8 | 39	Myb/SANT-like DNA-binding domain	-
C_591836	7tm_7 (79/79)	3 → 56 (p=0.0000)	0 | 4.2 | 56	7tm Chemosensory receptor	GO:0016021 (integral component of membrane); GO:0050909 (sensory perception of taste)
C_581761	zf-C2H2_6 (359/525)<br>zf-met (310/525)<br>zf-C2H2 (303/525)<br>zf-C2H2_4 (244/525)<br>zf-AD (157/525)	44 → 96 (p=0.0000)	1 | 26.5 | 97	C2H2-type zinc finger<br>Zinc-finger of C2H2 type<br>Zinc finger, C2H2 type<br>C2H2-type zinc finger<br>Zinc-finger associated domain (zf-AD)	GO:0046872 (metal ion binding)<br>-<br>GO:0046872 (metal ion binding)<br>-<br>GO:0005634 (nucleus); GO:0008270 (zinc ion binding)
C_591789	Chitin_bind_4 (409/409)	26 → 72 (p=0.0000)	4 | 19.6 | 72	Insect cuticle protein	GO:0042302 (structural constituent of cuticle)
C_605228	Catalase (54/55)<br>Catalase-rel (35/55)	3 → 16 (p=0.0000)	1 | 2.8 | 16	Catalase<br>Catalase-related immune-responsive	GO:0004096 (catalase activity); GO:0020037 (heme binding); GO:0055114 (oxidation-reduction process)<br>-
C_617431	-	1 → 11 (p=0.0000)	0 | 0.7 | 11	-	-
C_618183	-	8 → 26 (p=0.0000)	0 | 10.4 | 66	-	-
C_618336	Asp (171/172)	5 → 18 (p=0.0000)	1 | 8.8 | 69	Eukaryotic aspartyl protease	-

C_569943	Gamma-thionin (17/17)	1 → 10 (p=0.0000)	0 | 0.9 | 10	Gamma-thionin family	-
C_596849	p450 (589/589)	29 → 54 (p=0.0000)	9 | 29.2 | 64	Cytochrome P450	GO:0005506 (iron ion binding); GO:0016705 (oxidoreductase activity, acting on paired donors, with incorporation or reduction of molecular oxygen); GO:0020037 (heme binding); GO:0055114 (oxidation-reduction process)
C_618493	zf-H2C2_5 (64/454)	19 → 40 (p=0.0000)	0 | 23.8 | 48	C2H2-type zinc-finger domain	-
C_618161	Dynein_heavy (306/474)<br>AAA_9 (268/474)<br>MT (267/474)<br>AAA_6 (259/474)<br>AAA_8 (256/474)<br>DHC_N2 (255/474)<br>AAA_7 (236/474)<br>DHC_N1 (154/474)<br>AAA_5 (147/474)	21 → 43 (p=0.0000)	8 | 22.6 | 58	Dynein heavy chain and region D6 of dynein motor<br>ATP-binding dynein motor region D5<br>Microtubule-binding stalk of dynein motor<br>Hydrolytic ATP binding site of dynein motor region D1<br>P-loop containing dynein motor region D4<br>Dynein heavy chain, N-terminal region 2<br>P-loop containing dynein motor region D3<br>Dynein heavy chain, N-terminal region 1<br>AAA domain (dynein-related subfamily)	GO:0003777 (microtubule motor activity); GO:0007018 (microtubule-based movement); GO:0030286 (dynein complex)<br>-<br>-<br>-<br>-<br>-<br>-<br>-<br>GO:0005524 (ATP binding); GO:0016887 (ATPase activity)
C_611965	Peptidase_S10 (96/96)	5 → 18 (p=0.0000)	2 | 4.7 | 18	Serine carboxypeptidase	GO:0004185 (serine-type carboxypeptidase activity); GO:0006508 (proteolysis)
C_601164	Ank_2 (694/735)<br>Ank_4 (275/735)<br>Ank (243/735)	43 → 70 (p=0.0000)	18 | 34.6 | 74	Ankyrin repeats (3 copies)<br>Ankyrin repeats (many copies)<br>Ankyrin repeat	-<br>-<br>GO:0005515 (protein binding)
C_618396	EcKinase (533/539)	27 → 49 (p=0.0000)	12 | 27.5 | 54	Ecdysteroid kinase	-
C_617921	-	17 → 35 (p=0.0000)	6 | 13.8 | 35	-	-
C_618491	Peptidase_M13_N (290/323)<br>Peptidase_M13 (272/323)	15 → 32 (p=0.0000)	8 | 15.2 | 32	Peptidase family M13<br>Peptidase family M13	GO:0006508 (proteolysis)<br>GO:0004222 (metalloendopeptidase activity); GO:0006508 (proteolysis)
C_615350	Peptidase_M17 (83/88)<br>Peptidase_M17_N (45/88)	4 → 14 (p=0.0000)	1 | 4.1 | 14	Cytosol aminopeptidase family, catalytic domain<br>Cytosol aminopeptidase family, N-terminal domain	GO:0004177 (aminopeptidase activity); GO:0005622 (intracellular); GO:0006508 (proteolysis)<br>GO:0004177 (aminopeptidase activity); GO:0005622 (intracellular); GO:0006508 (proteolysis)
C_616307	MFS_1 (60/75)	5 → 15 (p=0.0000)	1 | 3.6 | 15	Major Facilitator Superfamily	GO:0016021 (integral component of membrane); GO:0055085 (transmembrane transport)

C_617940	GST_C (143/205)<br>GST_N_3 (106/205)<br>GST_N (85/205)	10 → 23 (p=0.0000)	3 | 10.1 | 30	Glutathione S-transferase, C-terminal domain<br>Glutathione S-transferase, N-terminal domain<br>Glutathione S-transferase, N-terminal domain	-<br>GO:0005515 (protein binding)<br>GO:0005515 (protein binding)
C_606174	DUF229 (119/119)	4 → 13 (p=0.0001)	3 | 5.2 | 16	Protein of unknown function (DUF229)	-
C_618274	Ig_3 (165/330)<br>C2-set_2 (143/330)	12 → 25 (p=0.0001)	9 | 15.1 | 32	Immunoglobulin domain<br>CD80-like C2-set immunoglobulin domain	-<br>-
C_591748	zf-C2H2_4 (68/148)<br>zf-met (64/148)<br>zf-C2H2_6 (62/148)<br>zf-C2H2 (54/148)<br>zf-AD (41/148)	8 → 19 (p=0.0001)	1 | 7.3 | 22	C2H2-type zinc finger<br>Zinc-finger of C2H2 type<br>C2H2-type zinc finger<br>Zinc finger, C2H2 type<br>Zinc-finger associated domain (zf-AD)	-<br>-<br>GO:0046872 (metal ion binding)<br>GO:0046872 (metal ion binding)<br>GO:0005634 (nucleus); GO:0008270 (zinc ion binding)
C_615899	CAP (169/185)	8 → 19 (p=0.0001)	1 | 8.7 | 26	Cysteine-rich secretory protein family	-
C_618518	PBP_GOBP (5/25)	2 → 9 (p=0.0001)	0 | 1.3 | 9	PBP/GOBP family	GO:0005549 (odorant binding)
C_613837	Ig_3 (13/82)	2 → 9 (p=0.0001)	0 | 2.8 | 9	Immunoglobulin domain	-
C_607490	COesterase (713/713)	41 → 62 (p=0.0002)	14 | 34.7 | 102	Carboxylesterase family	-
C_596600	Trypsin (75/75)	3 → 10 (p=0.0003)	0 | 3.8 | 19	Trypsin	GO:0004252 (serine-type endopeptidase activity); GO:0006508 (proteolysis)
C_616805	VWA (26/43)<br>VIT (26/43)	3 → 10 (p=0.0003)	0 | 2.1 | 12	von Willebrand factor type A domain<br>Vault protein inter-alpha-trypsin domain	-<br>-
Ir_group_B		21 → 36 (p=0.0003)	0 | 10.4 | 44		
C_611287	Ion_trans (136/172)<br>Ank_2 (128/172)<br>Ank (49/172)	11 → 22 (p=0.0004)	3 | 8.1 | 22	Ion transport protein<br>Ankyrin repeats (3 copies)<br>Ankyrin repeat	GO:0005216 (ion channel activity); GO:0006811 (ion transport); GO:0016020 (membrane); GO:0055085 (transmembrane transport)<br>-<br>GO:0005515 (protein binding)
C_617619	fn3 (14/22)	2 → 8 (p=0.0004)	0 | 1.2 | 8	Fibronectin type III domain	GO:0005515 (protein binding)
C_618664	DOMON (22/28)	2 → 8 (p=0.0004)	0 | 1.4 | 8	DOMON domain	-
C_605606	Glyco_hydro_30 (51/52)<br>Glyco_hydro_30C (44/52)	2 → 8 (p=0.0004)	0 | 2.6 | 8	Glycosyl hydrolase family 30 TIM-barrel domain<br>Glycosyl hydrolase family 30 beta sandwich domain	-<br>-
C_615614	Trehalose_recp (93/95)	5 → 13 (p=0.0004)	0 | 4.9 | 13	Trehalose receptor	GO:0008527 (taste receptor activity); GO:0016021 (integral component of membrane); GO:0050912 (detection of chemical stimulus involved in sensory perception of taste)

C_613870	Sulfotransfer_1 (157/165)	7 → 16 (p=0.0005)	2 | 6.6 | 16	Sulfotransferase domain	GO:0008146 (sulfotransferase activity)
C_618457	C_tripleX (43/123)	7 → 16 (p=0.0005)	3 | 6.1 | 16	Cysteine rich repeat	-
C_618509	Ig_3 (236/467)<br>V-set (136/467)	21 → 35 (p=0.0006)	13 | 22.6 | 35	Immunoglobulin domain<br>Immunoglobulin V-set domain	-<br>-
C_617687	C2-set_2 (100/265)	12 → 23 (p=0.0006)	7 | 12.6 | 23	CD80-like C2-set immunoglobulin domain	-
C_606481	adh_short (499/628)	29 → 45 (p=0.0006)	17 | 29.0 | 45	short chain dehydrogenase	-
C_611435	ABC_tran (546/610)<br>ABC2_membrane (296/610)	25 → 39 (p=0.0014)	16 | 25.7 | 39	ABC transporter<br>ABC-2 type transporter	GO:0005524 (ATP binding); GO:0016887 (ATPase activity)<br>GO:0016020 (membrane)
C_617772	-	1 → 5 (p=0.0015)	0 | 1.5 | 5	-	-
C_618391	p450 (8/20)	1 → 5 (p=0.0015)	0 | 1.0 | 7	Cytochrome P450	GO:0005506 (iron ion binding); GO:0016705 (oxidoreductase activity, acting on paired donors, with incorporation or reduction of molecular oxygen); GO:0020037 (heme binding); GO:0055114 (oxidation-reduction process)
C_617101	Exo_endo_phos_2 (2/18)	1 → 5 (p=0.0015)	0 | 0.9 | 12	Endonuclease-reverse transcriptase	-
C_616424	Alpha-amylase (173/195)	9 → 18 (p=0.0015)	4 | 9.6 | 20	Alpha amylase, catalytic domain	GO:0003824 (catalytic activity); GO:0005975 (carbohydrate metabolic process)
C_617758	JHBP (425/429)	26 → 40 (p=0.0018)	10 | 21.5 | 43	Haemolymph juvenile hormone binding protein (JHBP)	-
C_616414	cNMP_binding (162/261)<br>Ion_trans (129/261)	12 → 22 (p=0.0018)	8 | 12.5 | 22	Cyclic nucleotide-binding domain<br>Ion transport protein	-<br>GO:0005216 (ion channel activity); GO:0006811 (ion transport); GO:0016020 (membrane); GO:0055085 (transmembrane transport)
C_612239	Lig_chan (13/72)	6 → 13 (p=0.0021)	0 | 3.8 | 14	Ligand-gated ion channel	GO:0004970 (ionotropic glutamate receptor activity); GO:0016020 (membrane)
C_617756	Glyco_hydro_1 (163/165)	11 → 20 (p=0.0022)	0 | 8.1 | 50	Glycosyl hydrolase family 1	GO:0004553 (hydrolase activity, hydrolyzing O-glycosyl compounds); GO:0005975 (carbohydrate metabolic process)
C_618214	Serpin (268/271)	14 → 24 (p=0.0022)	6 | 12.7 | 29	Serpin (serine protease inhibitor)	-
C_592784	p450 (52/54)	3 → 8 (p=0.0032)	0 | 2.8 | 13	Cytochrome P450	GO:0005506 (iron ion binding); GO:0016705 (oxidoreductase activity, acting on paired donors, with incorporation or reduction of molecular oxygen); GO:0020037 (heme binding); GO:0055114 (oxidation-reduction process)

C_616701	Pkinase (19/45)<br>Pkinase_C (15/45)	3 → 8 (p=0.0032)	0 | 2.2 | 9	Protein kinase domain<br>Protein kinase C terminal domain	GO:0004672 (protein kinase activity); GO:0005524 (ATP binding); GO:0006468 (protein phosphorylation)<br>GO:0004674 (protein serine/threonine kinase activity); GO:0005524 (ATP binding); GO:0006468 (protein phosphorylation)
C_616314	Abhydro_lipase (252/368)<br>Abhydrolase_1 (101/368)	13 → 22 (p=0.0043)	3 | 17.3 | 36	Partial alpha/beta-hydrolase lipase region<br>alpha/beta hydrolase fold	GO:0006629 (lipid metabolic process)<br>-
C_607792	Peptidase_C1 (200/204)<br>Inhibitor_I29 (126/204)	10 → 18 (p=0.0044)	6 | 9.7 | 27	Papain family cysteine protease<br>Cathepsin propeptide inhibitor domain (I29)	GO:0006508 (proteolysis); GO:0008234 (cysteine-type peptidase activity)<br>-
C_618487	zf-met (19/61)<br>zf-Di19 (16/61)	2 → 6 (p=0.0049)	0 | 3.1 | 6	Zinc-finger of C2H2 type<br>Drought induced 19 protein (Di19), zinc-binding	-<br>-
C_618232	ASC (15/15)	2 → 6 (p=0.0049)	0 | 0.8 | 6	Amiloride-sensitive sodium channel	GO:0005272 (sodium channel activity); GO:0006814 (sodium ion transport); GO:0016020 (membrane)
desat_A2		2 → 6 (p=0.0049)	1 | 2.5 | 9		
C_615782	CBM_14 (340/340)	14 → 23 (p=0.0058)	6 | 16.6 | 49	Chitin binding Peritrophin-A domain	GO:0005576 (extracellular region); GO:0006030 (chitin metabolic process); GO:0008061 (chitin binding)
C_615705	F-box-like (47/62)	8 → 15 (p=0.0061)	0 | 3.2 | 17	F-box-like	GO:0005515 (protein binding)
C_613269	zf-C2HC (29/40)	1 → 4 (p=0.0063)	1 | 1.9 | 8	Zinc finger, C2HC type	GO:0003700 (transcription factor activity, sequence-specific DNA binding); GO:0005634 (nucleus); GO:0006355 (regulation of transcription, DNA-templated); GO:0008270 (zinc ion binding)
C_618552	-	1 → 4 (p=0.0063)	0 | 0.3 | 4	-	-
C_618575	SNF (2/11)	1 → 4 (p=0.0063)	0 | 0.6 | 4	Sodium:neurotransmitter symporter family	GO:0005328 (neurotransmitter:sodium symporter activity); GO:0006836 (neurotransmitter transport); GO:0016021 (integral component of membrane)
C_614434	-	1 → 4 (p=0.0063)	0 | 0.3 | 4	-	-
C_602496	DUF4804 (26/28)	1 → 4 (p=0.0063)	0 | 1.5 | 4	Domain of unknown function (DUF4804)	-
C_618173	COX1 (24/40)	1 → 4 (p=0.0063)	0 | 1.9 | 14	Cytochrome C and Quinol oxidase polypeptide I	GO:0004129 (cytochrome-c oxidase activity); GO:0005506 (iron ion binding); GO:0009055 (electron carrier activity); GO:0009060 (aerobic respiration); GO:0016021 (integral component of membrane); GO:0020037 (heme binding); GO:0055114 (oxidation-reduction process)
C_611283		1 → 4 (p=0.0063)	0 | 0.7 | 4		

C_586207	zf-C2H2 (188/376)	27 → 39 (p=0.0064)	0 | 18.6 | 39	Zinc finger, C2H2 type	GO:0046872 (metal ion binding)
	zf-met (143/376)			Zinc-finger of C2H2 type	-
	zf-C2H2_6 (135/376)			C2H2-type zinc finger	GO:0046872 (metal ion binding)
	zf-C2H2_4 (113/376)			C2H2-type zinc finger	-
C_616302	SSF (294/321)	15 → 24 (p=0.0076)	9 | 14.3 | 24	Sodium:solute symporter family	GO:0005215 (transporter activity); GO:0006810 (transport); GO:0016020 (membrane); GO:0055085 (transmembrane transport)
C_618475	zf-AD (101/105)	6 → 12 (p=0.0080)	3 | 4.9 | 12	Zinc-finger associated domain (zf-AD)	GO:0005634 (nucleus); GO:0008270 (zinc ion binding)
C_548938	zf-met (49/67)	6 → 12 (p=0.0080)	0 | 3.2 | 12	Zinc-finger of C2H2 type	-
	zf-C2H2 (49/67)			Zinc finger, C2H2 type	GO:0046872 (metal ion binding)
	zf-C2H2_4 (44/67)			C2H2-type zinc finger	-
	zf-C2H2_6 (36/67)			C2H2-type zinc finger	GO:0046872 (metal ion binding)

Table S7: Repeat content of the *Blattella germanica* and *Cryptotermes secundus* genomes.

	B. germanica		*C. secundus*	
Type	No. elements	Percentage	No. elements	Percentage
**class I - Retroelements**	2 773 999	34.53	2 312 964	36.58
SINEs	0	0.00	78	0.00
Penelope	9 721	0.13	10 339	0.23
LINEs	2 753 735	33.90	2 251 436	34.68
CRE/SLACS	0	0.00	4	0.00
L2/CR1/Rex	216 732	2.79	89 782	1.72
R1/LOA/Jockey	42 371	0.55	212 800	3.59
R2/R4/NeSL	14 628	0.23	639	0.02
RTE/Bov-B	2 224 205	26.94	1 706 647	25.95
L1/CIN4	904	0.02	252	0.00
LTR elements	20 264	0.64	61 450	1.90
BEL/Pao	12 727	0.36	474	0.02
Ty1/Copia	2 329	0.06	8 970	0.28
Gypsy/DIRS1	5 170	0.22	42 549	1.34
Retroviral	7	0.00	159	0.00
**class II - DNA transposons**	34 906	0.88	53 626	1.38
hobo-Activator	4 934	0.18	4 350	0.24
Tc1-IS630-Pogo	18 251	0.50	39 400	0.80
En-Spm	0	0.00	0	0.00
MuDR-IS905	0	0.00	0	0.00
PiggyBac	934	0.04	3 593	0.17
Tourist/Harbinger	6	0.00	210	0.01
Other	2	0.00	20	0.00
**Unclassified**	1 210 966	15.91	832 881	13.06
Small RNA	0	0.00	144	0.00
Satellites	0	0.00	53	0.00
Simple repeats	658 952	3.16	302 680	3.99
Low complexity	56 710	0.17	13 904	0.08
**Total interspersed repeats**		51.33		51.02
**Total non-interspersed repeats**		3.33		4.07
**Total repeats**		54.66		55.08

Table S8: Statistics of the association between repeat content in flanking regions, gene family expansion and species

parameter	χ^2	Δdf	*P*	No. obs	No. groups
gene family expansion	92.92	1	<0.0001	69 726	8 405
species	24 375.61	3	<0.0001	69 726	8 405
interaction	439.18	3	<0.0001	69 726	8 405

Table S9: Model parameters of the association between repeat content in flanking regions, gene family expansions and species. [1] compared to genes belonging to gene families that did not significantly change; [2] compared to *B. germanica*.

parameter	est ± se	df	*t*	*P*
expanded [1]	0.088 ± 0.009	61 317	9.639	$5.7e-22$
C. secundus[2]	0.119 ± 0.006	61 317	21.210	$< 1.0e-99$
Z. nevadensis[2]	-0.779 ± 0.006	61 317	-123.026	$< 1.0e-99$
M. natalensis[2]	-0.412 ± 0.006	61 317	-66.758	$< 1.0e-99$
B. germanica expanded [1]	0.010 ± 0.010	61 314	0.928	0.353
C. secundus expanded [1]	0.145 ± 0.025	61 314	5.685	$1.3e-08$
Z. nevadensis expanded [1]	0.245 ± 0.029	61 314	8.321	$8.9e-17$
M. natalensis expanded [1]	0.628 ± 0.030	61 314	20.816	$6.7e-96$

Table S10: Differentially expressed IRs

geneID	NvF/WvF	WvM	MvF
**Blattodea IRs**			
Bger_39798	-3.89		
Znev_04642	-	-	-2.60
Znev_15585	-	-	1.90
Znev_16376	-3.04	-	-4.23
Znev_18817	-	-6.14	5.85
Znev_18831	-2.38	-	-2.91
Znev_18854	-	-	3.40
Znev_18866	-3.22	-	-5.10
Znev_18895	-	-	1.63
Znev_18930	1.47	-	-
Znev_18945	-2.58	-	-3.43
Znev_18950	-3.36	-	-4.72
Znev_18974	-1.53	-	-1.43
Znev_18977	2.93	-	-
Znev_18985	-	-2.71	2.42
Znev_19004	-4.44	-	-4.80
Znev_19028	-	-	1.29
Znev_19037	2.63	-	3.13
Znev_19057	1.46	-	-
Csec_G04160	-2.07	-	-3.13
Csec_G04161	-1.50	-	-1.67
Csec_G04162	-2.26	-	-2.58
Csec_G10241	-1.70	-	-
Csec_G17212	-1.71	-	-1.10
Mnat_03961	4.94	1.48	3.26
**Group A IRs**			
Znev_01639	1.29	-	1.26
Znev_05745	-	-	1.12
Znev_18811	-1.75	-	-1.28
Znev_18812	-	-	1.23
Znev_18955	-	-	1.90
Znev_18956	-1.12	1.89	-3.01
Znev_18957	-3.51	4.79	-8.29
Znev_18958	-3.55	-	-5.27
Znev_18968	-1.35	-	-1.88
Znev_18989	4.09	2.60	-
Znev_19067	1.81	-	1.79
Mnat_14298	1.51	-	-
Mnat_16465	2.02	-	-
**Group B IRs**			
Znev_12219	-	-	1.85
Znev_18820	3.70	1.91	1.80
Znev_18825	-1.21	-	-
Znev_18826	1.21	-	1.14
Znev_18859	1.65	-	-
Znev_18919	-3.20	-	-3.63
Znev_18920	-	-2.12	1.15
Znev_18922	-2.41	-1.31	-1.10
Znev_18934	-3.77	4.12	-7.89
Znev_18935	-3.61	2.13	-5.74
Znev_18937	-3.37	-	-3.26
Znev_18938	-2.30	-	-2.25
Znev_18939	-2.67	-	-1.99
Znev_18941	1.24	-	-
Znev_18943	-	-3.72	3.06
Znev_18990	-	2.93	-2.45

geneID	NvF/WvF	WvM	MvF
Znev_19031	1.52	-	-
Znev_19050	-	-	2.05
Csec_G01746	1.62	-	-
Csec_G02881	-2.02	-	-
Csec_G02892	-	-	-1.70
Csec_G03872	-	-1.75	-
Csec_G06079	-	-1.89	-
Csec_G06973	3.31	2.29	1.02
Csec_G07625	-	-	-1.40
Csec_G09343	-	-	-2.58
Csec_G12192	-3.11	-	-3.80
Csec_G12571	-2.23	-	-2.93
Csec_G16315	-	-	0.97
Mnat_10145	1.98	-	-
Mnat_10147	-	2.01	-2.15
Mnat_14393	3.66	2.84	-
Mnat_14395	5.50	1.67	-
Mnat_15415	2.05	-	-
Mnat_15509	-	2.22	-
Mnat_15587	2.87	-	-
Mnat_16834	-2.27	-	-2.77
**Group C IRs**			
Znev_01873	2.20	-	2.45
Znev_01874	-	-	2.46
Znev_18835	-	-	1.31
Znev_18861	-2.33	-	-2.75
Csec_G02868	-	-	-1.67
Csec_G02869	-1.64	-	-1.34
Mnat_09267	-1.45	-	-1.46
**Group D IRs**			
Bger_40121	-3.25		
Znev_07227	2.08	-	1.61

Table S11: Differentially expressed ORs in *B. germanica* and termites

geneID	NvF/WvF	WvM	MvF
Bger_39217	-3.67	-	-
Znev_19048	-	-	-2.90
Znev_18836	-2.33	-	-3.95
Znev_18838	-	-	1.83
Znev_18841	-2.63	-	-2.70
Znev_18852	-	-2.52	3.01
Znev_19062	-3.19	-	-4.80
Znev_19064	-	-	-2.81
Znev_18855	-3.05	-	-3.17
Znev_18891	2.25	-	-
Znev_19069	-2.14	-	-
Znev_18908	-1.23	-	-
Znev_07294	-2.26	-	-
Znev_18972	1.84	-	-
Znev_18982	-2.44	-	-
Znev_18823	-	-3.23	2.60
Znev_19008	-	-	-2.36
Znev_19010	-2.84	-	-2.58
Znev_11756	1.50	-	-
Znev_19034	-	-	1.18
Znev_19038	-	-	1.42
Znev_19044	2.10	-	2.61
Csec_G07181	-3.00	-	-2.76
Csec_G08622	-1.66	-	-
Csec_G08633	-1.66	-1.63	-
Csec_G08635	-1.30	-	-1.74
Csec_G08666	-2.76	-	-1.58
Csec_G09183	-	-	-1.61
Csec_G09266	-3.15	-	-4.33
Csec_G09267	-2.21	-	-2.35
Csec_G09268	-2.61	-	-2.47
Csec_G09269	-	2.21	-2.44
Csec_G10644	2.20	1.97	-
Csec_G11043	-1.26	-	-
Csec_G16797	-2.71	-	-2.46
Csec_G16859	-	-1.36	-
Mnat_15635	-	-	1.47
Mnat_16988	5.82	-	4.08
Mnat_07594	-	-	-2.99
Mnat_09880	-	-	-1.05
Mnat_17263	4.46	2.27	-
Mnat_10995	-1.72	-	-1.35
Mnat_14693	1.78	2.37	-

Table S12: Desaturase occurrences in different species. Data for Blattodea and *L. migratoria*were analyzed in this project. Data for other species were taken from the literature[48] and only complete gene models considered.

Species	A1	A2	First				Ifc	Cyt-b5-r	Total
			B	C	D	E			
T. castaneum	2	9	1	1	1	1	1	2	18
D. melanogaster	3	1	1	0	1	1	1	2	10
N. vitripennis	2	1	8	4	0	1	1	2	19
A. mellifera	1	4	1	0	1	2	1	2	12
H. saltator	2	2	8	1	1	1	1	1	17
P. barbatus	1	3	4	0	1	1	1	1	12
L. migratoria	1	1	2	2	1	1	1	3	12
B. germanica	1	6	7	0	1	1	1	5	22
Z. nevadensis	2	2	3	1	1	1	1	2	13
C. secundus	2	2	3	1	1	1	1	3	14
M. natalensis	3	2	10	2	1	1	1	2	22

Table S13: Expression analysis of desaturases. Only values are shown where the p-value is < 0.05. The third gene of *C. secundus*belonging to subfamily B is split over scaffold boundaries and has therefore been named Csec-B-c-1 and Csec-B-c-2.

Family	name	geneID	NvF/WvF	WvM	MvF
Desat-A1	Bger-A1	Bger_17825	-4.02		
	Znev-A1-a	Znev_14773	1.63	-	1.24
	Znev-A1-b	Znev_05777	2.17	-	1.58
	Csec-A1-a	Csec_G08168	-	-	-
	Csec-A1-b	Csec_G13183	-	-	-
	Mnat-A1-a	Mnat_01743	-1.25	-	-
	Mnat-A1-b	Mnat_08601	-	-	-
	Mnat-A1-c	Mnat_10034	-1.29	-	-2.31
Desat-A2	Bger-A2-a	Bger_04328	-		
	Bger-A2-b	Bger_19901	-		
	Bger-A2-c	Bger_01068	-		
	Bger-A2-d	Bger_04054	5.91		
	Bger-A2-e	Bger_04065	-		
	Bger-A2-f	Bger_04057	4.00		
	Znev-A2-a	Znev_01237	2.10	-	1.95
	Znev-A2-b	Znev_19500	1.48	-	1.08
	Csec-A2-a	Csec_G02203	-	-	-
	Csec-A2-b	Csec_G02199	-	-	-1.46
	Mnat-A2-a	Mnat_12402	2.76	-	2.39
	Mnat-A2-b	Mnat_12398	-	-	-
Desat-B	Bger-B-a	Bger_17826	-7.04		
	Bger-B-b	Bger_00580	-		
	Bger-B-c	Bger_00584	2.41		
	Bger-B-d	Bger_12825	2.07		
	Bger-B-e	Bger_31359	3.61		
	Bger-B-f	Bger_00585	4.20		
	Bger-B-g	Bger_00586	-		
	Znev-B-a	Znev_14774	-1.92	-3.77	1.85
	Znev-B-b	Znev_05879	2.48	-	2.51
	Znev-B-c	Znev_16699	3.49	1.40	2.10
	Csec-B-a	Csec_G13185	-3.39	-	-3.49
	Csec-B-b	Csec_G08240	-	-	-
	Csec-B-c-1	Csec_G19111	-	-1.19	-
	Csec-B-c-2	Csec_G15733	-	-1.16	-
	Mnat-B-a	Mnat_01742	-	1.74	-2.49
	Mnat-B-b	Mnat_17019	5.30	-	-
	Mnat-B-c	Mnat_10836	7.24	2.34	4.41
	Mnat-B-d	Mnat_08598	-	-	-
	Mnat-B-e	Mnat_17410	-	-	3.91
	Mnat-B-f	Mnat_16495	5.47	2.42	-

Family	name	geneID	NvF/WvF	WvM	MvF
	Mnat-B-g	Mnat_09158	-	-	-
	Mnat-B-h	Mnat_08597	2.31	2.39	-
	Mnat-B-i	Mnat_18116	3.08	-	-
	Mnat-B-j	Mnat_18117	-	-	-
Desat-C	Znev-C	Znev_16655	-	-	-
	Csec-C	Csec_G13180	-	-1.57	1.47
	Mnat-C-a	Mnat_18100	-	-	-
	Mnat-C-b	Mnat_18101	-	2.15	-
Desat-D	Bger-D	Bger_04049	-		
	Znev-D	Znev_15801	-	-	-
	Csec-D	Csec_G02201	-	-	-
	Mnat-D	Mnat_12401	-	-	-
Desat-E	Bger-E	Bger_04061	-		
	Znev-E	Znev_01236	2.25	-	2.23
	Csec-E	Csec_G02204	-	-1.00	1.52
	Mnat-E	Mnat_12403	3.97	2.54	-
Desat-IFC	Bger-Ifc	Bger_10160	-1.37		
	Znev-Ifc	Znev_05247	-	-1.40	0.89
	Csec-Ifc	Csec_G02980	-	-	-
	Mnat-Ifc	Mnat_15445	-	-	-1.24
Desat-Cyt-b5-r	Bger-Cyt-b5-r-a	Bger_16012	-		
	Bger-Cyt-b5-r-b	Bger_16013	1.60		
	Bger-Cyt-b5-r-c	Bger_16793	-1.47		
	Bger-Cyt-b5-r-d	Bger_16794	-1.97		
	Bger-Cyt-b5-r-e	Bger_28499	-1.81		
	Znev-Cyt-b5-r-a	Znev_01408	-2.17	-2.88	-
	Znev-Cyt-b5-r-b	Znev_18432	1.99	-	2.07
	Csec-Cyt-b5-r-a	Csec_G14937	-	-1.54	-
	Csec-Cyt-b5-r-b	Csec_G14938	-	-1.54	2.29
	Csec-Cyt-b5-r-c	Csec_G06076	-2.76	-	-1.50
	Mnat-Cyt-b5-r-b	Mnat_04712	2.79	4.15	-
	Mnat-Cyt-b5-r-a	Mnat_15043	-	-	-

Table S14: Elongase gene tree in Blattodea. Bootstrap values > 70 are shown. The table lists the differential expression patterns between nymphs and females (NvF)/workers and females (WvF), workers and males (WvM), and males versus females (MvF). Values represent log2-fold change where p<0.05. Non significant differences are indicated with "-". Cells where data was unavailable are left blank.

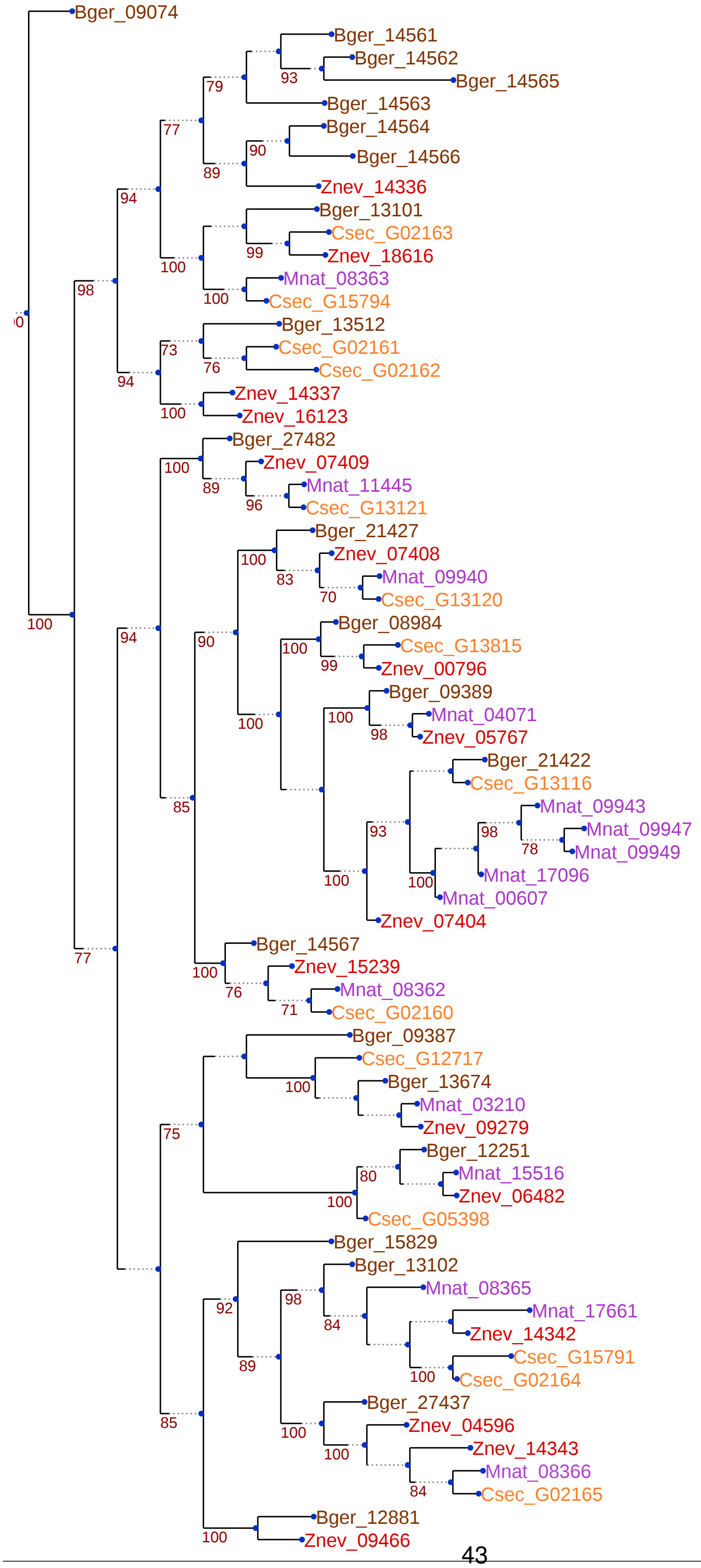

Elongase gene tree	NvF/WvF	WvM	MvF
Bger_09074	-		
Bger_14561	-3.94		
Bger_14562	-		
Bger_14565	-		
Bger_14563	-		
Bger_14564	-		
Bger_14566	-		
Znev_14336	2.26	-	1.76
Bger_13101	-		
Csec_G02163	3.00	-	2.62
Znev_18616	1.88	-	2.06
Mnat_08363	4.51	-	-
Csec_G15794	-	-	-
Bger_13512	-2.62		
Csec_G02161	-	-	1.34
Csec_G02162	2.25	-	1.86
Znev_14337	1.59	-	1.67
Znev_16123	2.15	-	1.43
Bger_27482	6.30		
Znev_07409	3.52	2.00	1.53
Mnat_11445	-	-	-
Csec_G13121	-	-	1.67
Bger_21427	4.92		
Znev_07408	2.58	-	-
Mnat_09940	-	1.44	-
Csec_G13120	-	-	1.93
Bger_08984	-		
Csec_G13815	-	-	-
Znev_00796	1.12	-	1.84
Bger_09389	-		
Mnat_04071	-	1.21	-
Znev_05767	-	-	-1.16
Bger_21422	-		
Csec_G13116	-	-	-
Mnat_09943	1.57	1.68	-
Mnat_09947	-1.30	-	-
Mnat_09949	2.20	1.89	-
Mnat_17096	2.84	-	-
Mnat_00607	2.88	-	-
Znev_07404	-	-	-
Bger_14567	1.71		
Znev_15239	-	-	-
Mnat_08362	3.49	-	-
Csec_G02160	-	-	1.34
Bger_09387	-		
Csec_G12717	-	-	-
Bger_13674	5.25		
Mnat_03210	1.72	1.77	-
Znev_09279	-	-	-
Bger_12251	-		
Mnat_15516	1.61	-	-
Znev_06482	-	-	-
Csec_G05398	-	-	-
Bger_15829	-3.87		
Bger_13102	4.13		
Mnat_08365	2.77	-	-
Mnat_17661	2.99	-	-
Znev_14342	-	-	-
Csec_G15791	-	-	-
Csec_G02164	-	-	-
Bger_27437	-		
Znev_04596	-	-2.55	1.93
Znev_14343	-1.36	-3.45	2.09
Mnat_08366	1.56	1.95	-
Csec_G02165	-	-	1.20
Bger_12881	-2.23		
Znev_09466	-	-	-

Table S15: Differential expression of CYP4G1 found in *B. germanica* and the three termites

Gene	geneID	NvF/WvF	WvM	MvF
CYP4G1	Bger_09844	-1.23		
	Csec_G03439	-	-	1.30
	Znev_05398	1.79	-	2.23
	Mnat_06635	4.15	-	-
	Mnat_06638	3.87	2.37	-
	Mnat_06640	3.96	2.97	-

Table S16: GO enrichment of CpG depletion-enrichment of the three termite species genes grouped(termite) and *B. germanica*. High CpG corresponds to the forth quartile whereas low CpG to the first quartile of CpG.

GO.ID	Term	Annotated	Significant	Expected	topgoFisher
Blattella germanica - high CpG					
GO:0006355	regulation of transcription, DNA-templat...	258	104	41.39	1e-30
GO:0050909	sensory perception of taste	422	115	67.7	2.9e-17
GO:0006313	transposition, DNA-mediated	39	23	6.26	7.4e-12
GO:0015074	DNA integration	56	26	8.98	4.2e-10
GO:0007186	G-protein coupled receptor signaling pat...	201	55	32.24	7.9e-09
GO:0006810	transport	797	124	127.86	1.8e-06
GO:0007223	Wnt signaling pathway, calcium modulatin...	10	8	1.6	2.1e-06
GO:0007275	multicellular organism development	101	27	16.2	8.3e-05
Blattella germanica - low CpG					
GO:0008152	metabolic process	2828	921	848.69	1e-30
GO:0005975	carbohydrate metabolic process	198	88	59.42	5.5e-13
GO:0055085	transmembrane transport	395	144	118.54	8.4e-12
GO:0006508	proteolysis	489	161	146.75	6.8e-11
GO:0055114	oxidation-reduction process	228	88	68.42	6.9e-09
GO:0006812	cation transport	180	75	54.02	4.6e-05
GO:0006281	DNA repair	44	21	13.2	6.7e-05
GO:0006457	protein folding	30	16	9	9.3e-05
Termites - high CpG					
GO:0007186	G-protein coupled receptor signaling pat...	489	246	93.66	1e-30
GO:0050909	sensory perception of taste	157	101	30.07	1e-30
GO:0006355	regulation of transcription, DNA-templat...	636	223	121.82	1e-30
GO:0015074	DNA integration	146	92	27.97	1e-30
GO:0007608	sensory perception of smell	126	72	24.13	9.8e-28
GO:0007275	multicellular organism development	162	76	31.03	4.9e-22
GO:0006810	transport	1727	359	330.8	9.4e-20
GO:0006030	chitin metabolic process	86	49	16.47	3.2e-19
GO:0007223	Wnt signaling pathway, calcium modulatin...	26	23	4.98	1.9e-16
GO:0055085	transmembrane transport	809	188	154.96	1.6e-12
GO:0009253	peptidoglycan catabolic process	20	14	3.83	4.8e-08
GO:0007156	homophilic cell adhesion via plasma memb...	67	28	12.83	1.2e-07
GO:0006313	transposition, DNA-mediated	16	12	3.06	1.4e-07
GO:0006334	nucleosome assembly	25	15	4.79	3.3e-07
GO:0007218	neuropeptide signaling pathway	15	10	2.87	8.7e-06
GO:0006508	proteolysis	892	174	170.86	1.1e-05
Termites - low CpG					
GO:0008152	metabolic process	7233	2090	2008.53	1e-30
GO:0055114	oxidation-reduction process	596	219	165.5	6.0e-19
GO:0055085	transmembrane transport	809	258	224.65	1.4e-13
GO:0006508	proteolysis	892	267	247.7	2.5e-12
GO:0044763	single-organism cellular process	2409	648	668.96	6.9e-10
GO:0005975	carbohydrate metabolic process	417	142	115.8	9.4e-09
GO:0006790	sulfur compound metabolic process	25	18	6.94	3.7e-08
GO:0006418	tRNA aminoacylation for protein translat...	113	56	31.38	1.1e-07
GO:0044267	cellular protein metabolic process	1905	549	529	2.9e-07
GO:0006886	intracellular protein transport	207	82	57.48	3.2e-07
GO:0006281	DNA repair	126	60	34.99	3.6e-07
GO:0019751	polyol metabolic process	19	14	5.28	8.2e-07
GO:0006635	fatty acid beta-oxidation	11	10	3.05	1.1e-06
GO:0006812	cation transport	317	102	88.03	8.0e-06
GO:0006419	alanyl-tRNA aminoacylation	11	9	3.05	2.2e-05
GO:0006468	protein phosphorylation	608	165	168.84	2.3e-05
GO:0006506	GPI anchor biosynthetic process	31	17	8.61	2.3e-05
GO:0007018	microtubule-based movement	101	38	28.05	4.4e-05
GO:0006259	DNA metabolic process	1146	265	318.23	4.4e-05
GO:0030001	metal ion transport	169	49	46.93	6.3e-05
GO:0009156	ribonucleoside monophosphate biosyntheti...	153	55	42.49	6.8e-05
GO:0009220	pyrimidine ribonucleotide biosynthetic p...	19	12	5.28	7.0e-05
GO:0046132	pyrimidine ribonucleoside biosynthetic p...	19	12	5.28	7.0e-05
GO:0044237	cellular metabolic process	4878	1339	1354.57	7.9e-05
GO:0009058	biosynthetic process	2752	683	764.2	8.1e-05
GO:0051188	cofactor biosynthetic process	70	37	19.44	8.3e-05
GO:0006457	protein folding	90	34	24.99	9.9e-05

Table S17: Number of differentially expressed genes containing zinc finger, bHLH and bZIP domains in *B. germanica* and termites.

	Nymph-/ Worker-biased	Adult-/ Queen-biased	Non-biased	χ^2	*P*
B. germanica					
Total	1 174 (3.9%)	1 149 (3.8%)	27 807 (92.3%)		
ZF	22 (3.1%)	5 (0.7%)	677 (96.2%)	19.9	4.8x10^{-5}
bHLH	4 (8.9%)	2 (4.4%)	39 (86.7%)	3.1	0.2
bZIP	0	3 (16.7%)	15 (83.3%)	8.6	0.013
Z. nevadensis					
Total	2 703 (17.0%)	2 615 (16.5%)	10 548 (66.5%)		
ZF	45 (7.6%)	217 (36.8%)	327 (55.5%)	177.1	3.4x10^{-39}
bHLH	12 (24.5%)	10 (20.4%)	27 (55.1%)	3.0	0.2
bZIP	3 (15.0%)	5 (25.0%)	12 (60.0%)	1.1	0.6
C. secundus					
Total	504 (2.8%)	1 237 (6.8%)	16 495 (90.5%)		
ZF	8 (1.0%)	126 (15.4%)	685 (83.6%)	94.4	3.1x10^{-21}
bHLH	2 (4.3%)	2 (4.3%)	42 (91.3%)	0.8	0.7
bZIP	0 (0%)	2 (7.4%)	25 (92.6%)	0.8	0.7
M. natalensis					
Total	3 022 (18.5%)	1 894 (11.6%)	11 404 (69.9%)		
ZF	53 (11.4%)	89 (19.1%)	324 (69.5%)	44.8	1.9x10^{-10}
bHLH	8 (27.6%)	3 (10.3%)	18 (62.1%)	1.6	0.5
bZIP	2 (12.5%)	5 (31.3%)	9 (26.3%)	6.0	0.049

Table S18: Differential expression patterns in Halloween genes in *B. germanica* and the three termites.

Gene	geneID		NvF		WvF	WvM	MvF		WvF	WvM	MvF		WvF	WvM	MvF
Disembodied	CYP302A1	Bger_24331	-	Znev_08701	-	-	-	Csec_G00785	-	-	-	Mnat_05257	-1.45	-	-1.46
Phantom	CYP306A1	Bger_21433	-	Znev_00957	1.84	-	1.80	Csec_G15254	-	-	-	Mnat_14835	1.77	-	-
Spook	CYP307A1	Bger_04901	-	Znev_04417	-	1.84	-	Csec_G01803	-	-	-	Mnat_00722	-	-	-
Spook	CYP307A1	Bger_25648	-												
Shade	CYP314A1	Bger_13798	1.42	Znev_02808	-2.17	3.77	-5.94	Csec_G14594	-	-	-	Mnat_01181	-	-	-
Shadow	CYP315A1	Bger_09617	-	Znev_14659	-2.80	-	-3.67	Csec_G17028	-	1.60	-2.04	Mnat_17585	-	-	-
Shadow	CYP315A1											Mnat_17645	-	-	-
Neverland		Bger_22030	-	Znev_04416	-1.24	1.14	-2.39	Csec_G01804	-1.20	-	-2.00	Mnat_00723	3.14	-	-

Table S19: Differentially expressed ecdysone kinases

geneID	NvF/WvF	WvM	MvF
Bger_00622	-1.00	-	-
Bger_06999	-1.34	-	-
Bger_07357	-2.13	-	-
Bger_07363	-2.91	-	-
Bger_07364	-2.89	-	-
Bger_07366	-2.47	-	-
Bger_09359	-2.10	-	-
Bger_09361	-2.01	-	-
Bger_09362	-2.70	-	-
Bger_09363	-3.64	-	-
Bger_09365	-3.35	-	-
Bger_14468	-2.81	-	-
Bger_19074	-1.31	-	-
Bger_23421	-1.58	-	-
Bger_25882	-2.68	-	-
Bger_25883	-2.01	-	-
Znev_00042	-	-	1.00
Znev_00081	1.96	-	1.81
Znev_04406	3.36	-	2.58
Znev_05651	3.54	-	2.73
Znev_05652	2.69	-	3.13
Znev_08648	5.82	-	5.28
Znev_08649	5.83	-	4.99
Znev_08650	1.21	-	1.87
Znev_11014	1.45	-	-
Znev_11017	1.56	-	1.48
Znev_13563	1.53	-	1.54
Znev_13564	2.29	-	1.28
Znev_16523	2.05	-	1.68
Znev_17283	3.23	-	3.47
Csec_G00058	-	-1.24	1.19
Csec_G01844	1.60	-	1.14
Csec_G12438	2.65	1.97	-
Csec_G12439	1.72	-	-
Csec_G12794	2.32	-	1.82
Mnat_01392	2.89	1.17	-
Mnat_01485	2.37	3.34	-
Mnat_03456	1.51	-2.68	4.16
Mnat_07555	2.34	-	-
Mnat_10201	3.64	-	3.02
Mnat_12486	4.94	-	-
Mnat_14878	3.56	1.99	-
Mnat_17088	4.68	3.72	-
Mnat_17089	2.64	2.07	-
Mnat_17595	4.23	3.31	-
Mnat_17702	3.58	-	-

Table S20: Differentially expressed JHBPs

geneID	NvF/WvF	WvM	MvF
Bger_00607	6.29	-	-
Bger_00608	-1.63	-	-
Bger_02147	-2.69	-	-
Bger_04597	-1.88	-	-
Bger_04598	-3.50	-	-
Bger_04599	-3.75	-	-
Bger_04719	-1.59	-	-
Bger_04721	3.55	-	-
Bger_08558	5.12	-	-
Bger_08560	2.94	-	-
Bger_08561	-3.76	-	-
Bger_08562	-2.24	-	-
Bger_08563	9.04	-	-
Bger_08564	-7.62	-	-
Bger_08711	-1.48	-	-
Bger_10169	7.65	-	-
Bger_10171	-3.43	-	-
Bger_12040	-1.07	-	-
Bger_20739	-1.33	-	-
Bger_20878	7.47	-	-
Bger_26455	5.47	-	-
Znev_02016	1.97	-	1.85
Znev_02017	2.30	-	2.19
Znev_02018	-	-1.70	2.19
Znev_02019	-	-1.52	1.96
Znev_02020	3.24	-	2.55
Znev_02021	1.64	-	1.69
Znev_02339	1.81	-	-
Znev_03421	1.63	-	1.33
Znev_03423	1.26	-	-
Znev_03425	3.70	1.53	2.17
Znev_03426	1.57	-	1.85
Znev_03427	3.33	-	2.36
Znev_03430	1.97	-	1.50
Znev_10019	4.26	2.31	1.95
Znev_10021	5.70	3.44	2.26
Znev_12339	3.48	3.05	-
Znev_14396	-2.90	-3.16	-
Znev_14457	3.74	-	2.85
Znev_15101	-	-3.09	2.35
Znev_15102	-	-1.67	2.10
Znev_16187	-	-2.24	2.65
Csec_G02970	-	-1.83	2.04
Csec_G02971	3.97	3.98	-
Csec_G04323	2.46	2.15	-
Csec_G06194	-	-	1.03
Csec_G07835	-	-1.44	1.53
Csec_G07893	-	-	1.36
Csec_G07894	-	-	1.74
Csec_G07895	-	-1.63	-
Csec_G07896	-1.42	-1.77	-
Csec_G07898	3.11	2.88	-
Csec_G07909	1.25	-	-
Csec_G07911	-	-2.06	1.19
Csec_G07912	-1.55	-	-
Mnat_08252	-	-	2.10
Mnat_10292	3.53	2.99	-

geneID	NvF/WvF	WvM	MvF
Mnat_10293	5.91	1.61	3.92
Mnat_10295	1.94	-	-
Mnat_10297	1.79	-	1.21
Mnat_10298	1.40	-	-
Mnat_10299	3.81	2.16	-
Mnat_10301	-1.42	-	-
Mnat_10546	-3.37	-	-3.78
Mnat_11632	6.03	-	4.32
Mnat_16285	2.66	-	2.87
Mnat_16286	2.34	-	2.54
Mnat_16976	7.29	7.47	-
Mnat_17338	1.34	-	-
Mnat_17339	-3.00	-4.27	1.68
Mnat_17918	-1.97	-	-

Table S21: Sequencing data for *B. germanica* and *C. secundus*.

**Species**<br>Library type	Read length (bp)	Raw data (Mb)	Raw sequence depth (x)
Blattella germanica			
paired-end - 180bp	100	150 100	75.05
paired-end - 500bp	100	62 100	31.05
mate pair - 3kb	100	139 400	69.7
mate pair - 8kb	100	60 900	30.45
**Total**		412 500	206
Cryptotermes secundus			
paired-end - 250bp	150	21 604	16.61
paired-end - 500bp	100	35 454	27.26
paired-end - 800bp	100	14 205	10.92
mate pair - 2kb	49	17 265	13.28
mate pair - 5kb	49	17 818	13.70
mate pair - 10kb	49	16 124	12.40
**Total**		122 470	94.17

Table S22: Source websites and version numbers of 20 arthropod genomes and proteomes that were used for comparative analyses. *: gene models of key gene families were manually improved. †: species is included in the analysis of chemosensory receptors.

species	source website	data files
P. barbatus†	http://hymenopteragenome.org	genome 1.0, gff 1.2 (fixed)
D. melanogaster†	ftp://ftp.flybase.net/releases/FB2016_04/	CDS 6.12
H. saltator†	http://hymenopteragenome.org	CDS 3.3
R. prolixus†	ftp://ftp.ensemblgenomes.org/pub/metazoa/release-32/	genome RproC1, gff 1.32
T. castaneum†	https://www.ncbi.nlm.nih.gov/genome/216	genome 5.2, gff 5
A. mellifera†	ftp://ftp.ensemblgenomes.org/pub/metazoa/release-32/	genome 4.5, gff 4.5
E. danica†	ftp://ftp.hgsc.bcm.edu/I5K-pilot/Mayfly/genome_assemblies/	CDS 0.5.3
L. migratoria†	http://159.226.67.243/download.htm	genome 2.4.1, gff 2.4.1
N. vitripennis†	http://arthropods.eugenes.org/EvidentialGene/nasonia/genes/	CDS 2.1
P. canadensis	https://www.ncbi.nlm.nih.gov/genome/?term=txid91411	GCF_001313835.1 ASM131383v1
A. cephalotes	http://hymenopteragenome.org	genome 1.0, gff 1.2
C. floridanus	http://hymenopteragenome.org	genome 3.3, gff 3.3
L. humile	http://hymenopteragenome.org	genome 1.0, gff 1.2
S. invicta	http://hymenopteragenome.org	genome 1.0, gff 2.2.3
A. echinatior	http://hymenopteragenome.org	CDS 3.8
A. aegypti	ftp://ftp.ensemblgenomes.org/pub/metazoa/release-32/	CDS 3
S. maritima	ftp://ftp.ensemblgenomes.org/pub/metazoa/release-32/	CDS Smar1
*M. natalensis**†	http://gigadb.org/dataset/100057	genome 1, gff 1.2
*Z. nevadensis**†	http://termitegenome.org	genome 1.0, gff 2.2
*C. secundus**†	see table **??**	genome 1.0, gff 1.0
*B. germanica**†	see table **??**	genome 1.0, gff 1.0

Table S23: Protein sequences that were used to create Hidden Markov Models of two protein regions used to identify IRs. An HMM of the C-terminal IR region that consists of the ligand-binding domain (S1,S2) and the ion channel was created from **(a)**. An HMM of the amino-terminal domain that is found in iGluRs and IR8a/IR25a was created from **(b)**. *Z. nevadensis* sequences were taken from,[37] the remaining sequences were taken from.[35] Full species names: *Bombyx mori*, *Lottia gigantea*, *Aplysia californica*, *Ceratitis capitata*, *Anopheles gambiae*, *Culex quinquefasciatus*, *Pediculus humanus*, *Acyrthosiphon pisum*, *Daphnia pulex*.

A. aegypti	***B. mori***	***L. gigantea***
AaegIR87a.2	BmorIR87a	LgigIR256
AaegIR7s.1	BmorIR143	LgigIR275
AaegIR41p.1	BmorIR75d	LgigIR287
AaegIR116	BmorIR40a	LgigIR290
AaegIR7s.2	BmorIR76b	LgigIR25a
A. californica	***C. capitata***	***N. vitripennis***
AcalIR209	CcapIR230	NvitIR68a
AcalIR212	CcapIR249	NvitIR75u.1
AcalIR214	CcapIR251	NvitIR21a
AcalIR216	CcapIR260	NvitIR8a
AcalIR217	CcapIR261.1	NvitIR25a
A. gambiae	***C. quinquefasciatus***	***P. humanus***
AgamIR100a	CquiIR7h	PhumIR41a
AgamIR7y	CquiIR75l	PhumIR68a
AgamIR75h.1	CquiIR41m	PhumIR75d
AgamIR142	CquiIR92c	PhumIR93a
AgamIR60a	CquiIR125.2	PhumIR25a
A. mellifera	***D. melanogaster***	***T. castaneum***
AmelIR218	DmelIR25a	TcasIR40a
AmelIR68a	DmelIR8a	TcasIR76b
AmelIR93a	DmelIR75a	TcasIR25a
AmelIR8a	DmelIR68b	TcasIR144
AmelIR75f.1	DmelIR94d	TcasIR100l
A. pisum	***D. pulex***	***Z. nevadensis***
ApisIR21a	DpulIR25a	ZnevIR25a
ApisIR76b	DpulIR302	ZnevIR75o
ApisIR25a	DpulIR148	ZnevIR142
ApisIR8a	DpulIR165	ZnevIR196
ApisIR75d.2	DpulIR188	ZnevIR202
C. elegans		
CeleIR264		

(a) Selection of ligand-specific IR proteins.

D. melanogaster	***A. pisum***
DmelGluRIIC	ApisIR25a
DmelGluRIIA	
DmelGluRIIB	***B. mori***
DmelClumsy	BmorIR25a
DmelGluRIID	
DmelGluRIIE	***C. quinquefasciatus***
DmelGlu-R1	CquiIR25a
DmelGlu-R1B	CquiIR8a
DmelNmdar2	
DmelNmdar1	***D. pulex***
DmelIR8a	DpulIR25a
DmelIR25a	
	L. gigantea
A. aegypti	LgigIR25a
AaegIR8a	
AaegIR25a	***N. vitripennis***
	NvitNMDAR1
A. californica	NvitIR8a
AcalIR25a	NvitIR25a
AcalGluRK4	
	P. humanus
A. gambiae	PhumIR8a
AgamGLURIIc	PhumIR25a
AgamGLURIIb	
AgamGLURIIa	***T. castaneum***
AgamNMDAR1	TcasGluRK1
AgamGLURIId	TcasIR8a
AgamNMDAR3	TcasIR25a
AgamGLURI	
AgamIR25a	***Z. nevadensis***
AgamIR8a	ZnevNMDAR4
AgamNMDAR2	ZnevKAINATE1
AgamGLURIIe	ZnevKAINATE2
	ZnevKAINATE3
A. mellifera	ZnevKAINATE4
AmelIR25a	
AmelIR8a	

(b) Selection of iGluR and IR8a/IR25a proteins.

References

1. Gnerre, S. *et al.* High-quality draft assemblies of mammalian genomes from massively parallel sequence data. *Proceedings of the National Academy of Sciences* **108**, 1513–1518 (2011).

2. Martin, M. Cutadapt removes adapter sequences from high-throughput sequencing reads. *EMBnet.journal* **17**, pp. 10–12 (2011).

3. Fuchs, A., Heinze, J., Reber-Funk, C. & Korb, J. Isolation and characterization of six microsatellite loci in the drywood termite Cryptotermes secundus (Kalotermitidae). *Molecular Ecology Notes* **3**, 355–357 (2003).

4. Li, Y., Hu, Y., Bolund, L. & Wang, J. State of the art de novoassembly of human genomes from massively parallel sequencing data. *Human Genomics* **4**, 271 (2010).

5. Holt, C. & Yandell, M. MAKER2: an annotation pipeline and genome-database management tool for second-generation genome projects. *BMC Bioinformatics* **12**, 491 (2011).

6. Keller, O., Kollmar, M., Stanke, M. & Waack, S. A novel hybrid gene prediction method employing protein multiple sequence alignments. *Bioinformatics* **27**, 757–763 (2011).

7. Simão, F. A., Waterhouse, R. M., Ioannidis, P., Kriventseva, E. V. & Zdobnov, E. M. BUSCO: assessing genome assembly and annotation completeness with single-copy orthologs. *Bioinformatics* **31**, 3210–3212 (2015).

8. Borodovsky, M., Mills, R., Besemer, J. & Lomsadze, A. Prokaryotic Gene Prediction Using GeneMark and GeneMark.hmm. In *Current Protocols in Bioinformatics* (John Wiley & Sons, Inc., 2002). DOI: 10.1002/0471250953.bi0405s01.

9. Korf, I. Gene finding in novel genomes. *BMC Bioinformatics* **5**, 59 (2004).

10. Birney, E., Clamp, M. & Durbin, R. GeneWise and Genomewise. *Genome Research* **14**, 988–995 (2004).

11. Stanke, M. *et al.* AUGUSTUS: ab initio prediction of alternative transcripts. *Nucleic Acids Research* **34**, W435–W439 (2006).

12. Campbell, M. A., Haas, B. J., Hamilton, J. P., Mount, S. M. & Buell, C. R. Comprehensive analysis of alternative splicing in rice and comparative analyses with Arabidopsis. *BMC Genomics* **7**, 327 (2006).

13. Kong, L. *et al.* CPC: assess the protein-coding potential of transcripts using sequence features and support vector machine. *Nucleic Acids Research* **35**, W345–W349 (2007).

14. Min, X. J., Butler, G., Storms, R. & Tsang, A. OrfPredictor: predicting protein-coding regions in EST-derived sequences. *Nucleic Acids Research* **33**, W677–W680 (2005).

15. Elsik, C. G. *et al.* Creating a honey bee consensus gene set. *Genome Biology* **8**, R13 (2007).

16. Fischer, S. *et al.* Using OrthoMCL to assign proteins to OrthoMCL-DB groups or to cluster proteomes into new ortholog groups. *Current Protocols in Bioinformatics* **Chapter 6**, Unit 6.12.1–19 (2011).

17. Finn, R. D. *et al.* The Pfam protein families database: towards a more sustainable future. *Nucleic Acids Research* **44**, D279–D285 (2016).

18. Slater, G. S. C. & Birney, E. Automated generation of heuristics for biological sequence comparison. *BMC Bioinformatics* **6**, 31 (2005).

19. Notredame, C., Higgins, D. G. & Heringa, J. T-Coffee: A novel method for fast and accurate multiple sequence alignment. *Journal of Molecular Biology* **302**, 205–217 (2000).

20. Capella-Gutiérrez, S., Silla-Martínez, J. M. & Gabaldón, T. trimAl: a tool for automated alignment trimming in large-scale phylogenetic analyses. *Bioinformatics* **25**, 1972–1973 (2009).

21. Stamatakis, A. RAxML version 8: a tool for phylogenetic analysis and post-analysis of large phylogenies. *Bioinformatics* **30**, 1312–1313 (2014).

22. Darriba, D., Taboada, G. L., Doallo, R. & Posada, D. ProtTest 3: fast selection of best-fit models of protein evolution. *Bioinformatics* **27**, 1164–1165 (2011).

23. Huerta-Cepas, J., Serra, F. & Bork, P. ETE 3: Reconstruction, Analysis, and Visualization of Phylogenomic Data. *Molecular Biology and Evolution* **33**, 1635–1638 (2016).

24. Dohmen, E., Kremer, L. P. M., Bornberg-Bauer, E. & Kemena, C. DOGMA: domain-based transcriptome and proteome quality assessment. *Bioinformatics* **32**, 2577–2581 (2016).

25. Gurevich, A., Saveliev, V., Vyahhi, N. & Tesler, G. QUAST: quality assessment tool for genome assemblies. *Bioinformatics* **29**, 1072–1075 (2013).

26. Grabherr, M. G. *et al.* Full-length transcriptome assembly from RNA-Seq data without a reference genome. *Nature Biotechnology* **29**, 644–652 (2011).

27. Fu, L., Niu, B., Zhu, Z., Wu, S. & Li, W. CD-HIT: accelerated for clustering the next-generation sequencing data. *Bioinformatics* **28**, 3150–3152 (2012).

28. Kim, D. *et al.* TopHat2: accurate alignment of transcriptomes in the presence of insertions, deletions and gene fusions. *Genome Biology* **14**, R36 (2013).

29. Roberts, A., Trapnell, C., Donaghey, J., Rinn, J. L. & Pachter, L. Improving RNA-Seq expression estimates by correcting for fragment bias. *Genome Biology* **12**, R22 (2011).

30. Drost, H.-G., Gabel, A., Grosse, I. & Quint, M. Evidence for Active Maintenance of Phylotranscriptomic Hourglass Patterns in Animal and Plant Embryogenesis. *Molecular Biology and Evolution* **32**, 1221–1231 (2015).

31. Ferreira, P. G. *et al.* Transcriptome analyses of primitively eusocial wasps reveal novel insights into the evolution of sociality and the origin of alternative phenotypes. *Genome biology* **14**, R20 (2013).

32. Li, L., Stoeckert, C. J. & Roos, D. S. OrthoMCL: Identification of Ortholog Groups for Eukaryotic Genomes. *Genome Research* **13**, 2178–2189 (2003).

33. Eddy, S. R. Profile hidden Markov models. *Bioinformatics* **14**, 755–763 (1998).

34. Katoh, K. & Standley, D. M. MAFFT Multiple Sequence Alignment Software Version 7: Improvements in Performance and Usability. *Molecular Biology and Evolution* **30**, 772–780 (2013).

35. Croset, V. *et al.* Ancient Protostome Origin of Chemosensory Ionotropic Glutamate Receptors and the Evolution of Insect Taste and Olfaction. *PLOS Genetics* **6**, e1001064 (2010).

36. Drosophila 12 Genomes Consortium *et al.* Evolution of genes and genomes on the Drosophila phylogeny. *Nature* **450**, 203–218 (2007).

37. Terrapon, N. *et al.* Molecular traces of alternative social organization in a termite genome. *Nature Communications* **5**, 3636 (2014).

38. Krieger, J., Klink, O., Mohl, C., Raming, K. & Breer, H. A candidate olfactory receptor subtype highly conserved across different insect orders. *Journal of Comparative Physiology. A, Neuroethology, Sensory, Neural, and Behavioral Physiology* **189**, 519–526 (2003).

39. Price, M. N., Dehal, P. S. & Arkin, A. P. FastTree: Computing Large Minimum Evolution Trees with Profiles instead of a Distance Matrix. *Molecular Biology and Evolution* **26**, 1641–1650 (2009).

40. Letunic, I. & Bork, P. Interactive tree of life (iTOL) v3: an online tool for the display and annotation of phylogenetic and other trees. *Nucleic Acids Research* **44**, W242–245 (2016).

41. Yang, Z. PAML 4: Phylogenetic Analysis by Maximum Likelihood. *Molecular Biology and Evolution* **24**, 1586–1591 (2007).

42. Benton, R., Vannice, K. S., Gomez-Diaz, C. & Vosshall, L. B. Variant Ionotropic Glutamate Receptors as Chemosensory Receptors in Drosophila. *Cell* **136**, 149–162 (2009).

43. Loewenstein, Y., Portugaly, E., Fromer, M. & Linial, M. Efficient algorithms for accurate hierarchical clustering of huge datasets: tackling the entire protein space. *Bioinformatics* **24**, i41–i49 (2008).

44. Rappoport, N., Linial, N. & Linial, M. ProtoNet: charting the expanding universe of protein sequences. *Nature Biotechnology* **31**, 290–292 (2013).

45. Han, M. V., Thomas, G. W. C., Lugo-Martinez, J. & Hahn, M. W. Estimating Gene Gain and Loss Rates in the Presence of Error in Genome Assembly and Annotation Using CAFE 3. *Molecular Biology and Evolution* **30**, 1987–1997 (2013).

46. Simola, D. F. *et al.* Social insect genomes exhibit dramatic evolution in gene composition and regulation while preserving regulatory features linked to sociality. *Genome Research* **23**, 1235–1247 (2013).

47. Kapheim, K. M. *et al.* Genomic signatures of evolutionary transitions from solitary to group living. *Science* **348**, 1139–1143 (2015).

48. Helmkampf, M., Cash, E. & Gadau, J. Evolution of the insect desaturase gene family with an emphasis on social Hymenoptera. *Molecular Biology and Evolution* 456–471 (2015).
